# Forward and Pullback Dynamics of Nonautonomous Integrodifference Equations: Basic Constructions

**DOI:** 10.1007/s10884-020-09887-8

**Published:** 2020-09-02

**Authors:** Huy Huynh, Peter E. Kloeden, Christian Pötzsche

**Affiliations:** 1 Institut für Mathematik, Universität Klagenfurt, 9020 Klagenfurt, Austria; 2 Mathematisches Institut, Universität Tübingen, 72076 Tübingen, Germany

**Keywords:** Forward limit set, Forward attractor, Pullback attractor, Asymptotically autonomous equation, Integrodifference equation, Urysohn operator, 37C70, 37C60, 45G15, 92D40

## Abstract

In theoretical ecology, models describing the spatial dispersal and the temporal evolution of species having non-overlapping generations are often based on integrodifference equations. For various such applications the environment has an aperiodic influence on the models leading to nonautonomous integrodifference equations. In order to capture their long-term behaviour comprehensively, both pullback and forward attractors, as well as forward limit sets are constructed for general infinite-dimensional nonautonomous dynamical systems in discrete time. While the theory of pullback attractors, but not their application to integrodifference equations, is meanwhile well-established, the present novel approach is needed in order to understand their future behaviour.

## Introduction

Integrodifference equations not only occur as temporal discretisations of integrodifferential equations or as time-1-maps of evolutionary differential equations, but are of interest in themselves. First and foremost, they are a popular tool in theoretical ecology to describe the dispersal of species having non-overlapping generations (see, for instance, [18] or [9, 17, 25]). While the theory of Urysohn or Hammerstein integral equations is now rather classical [19], both numerically and analytically, our goal is here to study their iterates from a dynamical systems perspective. This means one is interested in the long term behaviour of recursions based on a fixed nonlinear integral operator. In applications, the iterates for instance represent the spatial distribution of interacting species over a habitat. One of the central questions in this context is the existence and structure of an attractor. These invariant and compact sets attract bounded subsets of an ambient state space *X* and fully capture the asymptotics of an autonomous dynamical system [8, 23]. The dynamics inside the attractor can be very complicated and even chaotic [7].

Extending this situation, the main part of this paper is devoted to general nonautonomous difference equations in complete metric spaces. Their right-hand side can depend on time allowing to model the dispersal of species in temporally fluctuating environments [3, 9] being not necessarily periodic. Thus, the behaviour depends on both the initial and the actual time. This is why many dynamically relevant objects are contained in the extended state space ${\mathbb {Z}}\times X$ (one speaks of nonautonomous sets) [10], rather than being merely subsets of the state space *X* as in the autonomous case. Furthermore, a complete description of the dynamics in a time-variant setting necessitates a strict distinction between forward and pullback convergence [10, 15]. For this reason only a combination of several attractor notions yields the full picture:

- The *pullback attractor* [4, 11, 15, 20] is a compact, invariant nonautonomous set which attracts all bounded sets from the past. As fixed target problem, it is based on previous information, at a fixed time from increasingly earlier initial times. Since it consists of bounded entire solutions to a nonautonomous system (see [20, p. 17, Cor. 1.3.4]), a pullback attractor can be seen as an extension of the global attractor to nonautonomous problems and apparently captures the essential dynamics to a certain point. Meanwhile the corresponding theory is widely developed in discrete and continuous time. However, pullback attractors reflect the past rather than the future of systems (see [14]) and easy examples demonstrate that differential or difference equations with identical pullback attractors might have rather different asymptotics as $t\rightarrow \infty$ and possibly feature limit sets, which are not captured by the pullback dynamics.
- This led to the development of *forward attractors*, which are also compact and invariant nonautonomous sets [15]. This dual concept depends on information from the future and given a fixed initial time, the actual time increases beyond all bounds — they are a moving target problem. Forward attractors are not unique, independent of pullback attractors, but often do not exist. Nevertheless, we will describe forward attractors using a pullback construction, even though this has the disadvantage that information on the system over the entire time axis ${\mathbb {Z}}$ is required.
- Therefore, it was suggested in [12] to work with *forward limit sets*, a concept related to the uniform attractor due to [24]. They correctly describe the asymptotic behaviour of all forward solutions to a nonautonomous difference equation. These limit sets have forward attraction properties, but different from pullback and forward attractors, they are not (even positively) invariant and constitute a single compact set, rather than a nonautonomous set. Nonetheless, asymptotic forms of positive (and negative) invariance do hold.

The situation for forward attractors and limit sets is not as well-established as their pullback counterparts and deserves to be developed for the above reasons. Their initial construction in [12, 16] requires a locally compact state space, but recent continuous-time results in [6], which extend these to infinite-dimensional dynamical systems, will be transferred here. We indeed address nonautonomous difference equations in (not necessarily locally compact) metric and Banach spaces, introduce the mentioned attractor types and study their properties.

This brings us to our second purpose. The above abstract setting allows concrete applications to a particularly interesting class of infinite-dimensional dynamical systems in discrete time, namely integrodifference equations (IDEs for short). We provide sufficient criteria for the existence of pullback attractors tailor-made for a quite general class of IDEs. Their right-hand sides go beyond pure integral operators and might also include superposition operators, which are used to describe populations having a sedentary fraction. Such results follow from a corresponding theory of set contractions contained in [20, pp. 15ff], [19, pp. 79ff]. For completely continuous right-hand sides (i.e., Urysohn operators) we construct forward limit sets and provide an application to asymptotically autonomous IDEs. We restrict to rather simple IDEs in the space of continuous functions over a compact domain as state space. More complicated equations and the behaviour of attractors under spatial discretisation will be tackled in future papers.

The contents of this paper are as follows: In Sect. 2 we establish the necessary terminology and provide a useful dissipativity condition for nonautonomous difference equations. The key notions related to pullback convergence, i.e., limit sets and attractors are reviewed and established in Sect. 3. The subsequent Sect. 4 addresses the corresponding notions in forward time. In detail, it establishes forward limit sets and their (weakened) invariance properties. For a class of asymptotically autonomous equations it is shown that their forward limit sets coincide with the global attractor of the limit equation. Moreover, a construction of forward attractors is suggested. Finally, in Sect. 5 we provide some applications to various IDEs. In particular, we illustrate the above theoretical results by studying pullback attractors and forward limit sets.

Notation Let ${\mathbb {R}}_+{:=}[0,\infty )$. A *discrete interval* ${\mathbb {I}}$ is defined as the intersection of a real interval with the integers ${\mathbb {Z}}$, ${\mathbb {I}}'{:=}\left\{ t\in {\mathbb {I}}:\,t+1\in {\mathbb {I}}\right\}$ and ${\mathbb {N}}_0{:=}\left\{ 0,1,2,\ldots \right\}$.

On a metric space (*X*, *d*), $I_X$ is the identity map, $B_r(x){:=}\left\{ y\in X:\,d(x,y)<r\right\}$ the open ball with center $x\in X$ and radius $r>0$, and $\bar{B}_r(0)$ denotes its closure. We write ${\text {dist}}\bigl (x,A\bigr ){:=}\inf _{a\in A}d(x,a)$ for the distance of *x* from a set $A\subseteq X$ and $B_r(A){:=}\left\{ x\in X:\,{\text {dist}}\bigl (x,A\bigr )<r\right\}$ for its *r*-neighbourhood. The *Hausdorff semidistance* of bounded and closed subsets $A,B\subseteq X$ is defined as

$$\begin{aligned} {\text {dist}}\bigl (A,B\bigr ){:=}\sup _{a\in A}\inf _{b\in B}d(a,b). \end{aligned}$$

The *Kuratowski measure of noncompactness* on *X* (cf. [19, pp. 16ff, I.5]) is denoted by $\chi :{\mathfrak {B}}(X)\rightarrow {\mathbb {R}}$, where ${\mathfrak {B}}(X)$ stands for the family of bounded subsets of *X*.

A mapping  is said to be *bounded*, if it maps bounded subsets of *X* into bounded sets and *globally bounded*, if  is bounded. We say a bounded  satisfies a *Darbo condition*, if there exists a real constant $k\ge 0$ such thatThe smallest such *k* is the *Darbo constant*  of . A *completely continuous* mapping  is bounded, continuous and satisfies .

A subset ${\mathcal {A}}\subseteq {\mathbb {I}}\times X$ with *t*-*fibres* ${\mathcal {A}}(t){:=}\{x\in X:\,(t,x)\in {\mathcal {A}}\}$, $t\in {\mathbb {I}}$, is called *nonautonomous set*. If all fibres ${\mathcal {A}}(t)\subseteq X$, $t\in {\mathbb {I}}$, are compact, then ${\mathcal {A}}$ is denoted as *compact* nonautonomous set and we proceed accordingly with other topological notions. Furthermore, one speaks of a *bounded* nonautonomous set ${\mathcal {A}}$, if there exists real $R>0$ and a point $x_0\in X$ such that ${\mathcal {A}}(t)\subseteq B_R(x_0)$ holds for all $t\in {\mathbb {I}}$.

Finally, on a Banach space *X*, *L*(*X*) denotes the space of bounded linear operators and  is the *spectral radius* of a .

## Nonautonomous Difference Equations

Unless otherwise noted, let (*X*, *d*) be a complete metric space. We consider nonautonomous difference equations in the abstract form 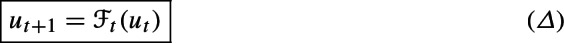 with continuous right-hand sides  and defined on closed sets $U_t\subseteq X$, $t\in {\mathbb {I}}'$. For an *initial time* $\tau \in {\mathbb {I}}$, a *forward solution* to $(\varDelta )$ is a sequence $(\phi _t)_{\tau \le t}$ with $\phi _t\in U_t$ satisfying2.1for all $\tau \le t$, $t\in {\mathbb {I}}'$, while an *entire solution* $(\phi _t)_{t\in {\mathbb {I}}}$ satisfies (2.1) on ${\mathbb {I}}'$. The unique forward solution starting at $\tau \in {\mathbb {I}}$ in $u_\tau \in U_\tau$ is denoted by $\varphi (\cdot ;\tau ,u_\tau )$; it is denoted as *general solution* to $(\varDelta )$ and reads as2.2as long as the compositions stay in $U_t$. Under the inclusion , $t\in {\mathbb {I}}'$, the general solution $\varphi (t;\tau ,\cdot ):U_\tau \rightarrow U_t$ exists for all $\tau \le t$ and the *process property*

2.3 $$\begin{aligned} \varphi (t;s,\varphi (s;\tau ,u))=\varphi (t;\tau ,u)\quad \text {for all }\tau \le s\le t,\,u\in U_\tau \end{aligned}$$

holds; we introduce the nonautonomous set ${\mathcal {U}}{:=}\left\{ (t,u)\in {\mathbb {I}}\times X:\,u\in U_t\right\}$.

One denotes $(\varDelta )$ as $\theta$-*periodic* with some $\theta \in {\mathbb {N}}$, if , $U_t=U_{t+\theta }$ and tacitly ${\mathbb {I}}={\mathbb {Z}}$ hold for all $t\in {\mathbb {Z}}$. In this case the general solution satisfies

2.4 $$\begin{aligned} \varphi (t+\theta ;\tau +\theta ,u_\tau )=\varphi (t;\tau ,u_\tau )\quad \text {for all }\tau \le t,\,(\tau ,u_\tau )\in {\mathcal {U}} \end{aligned}$$

yielding a rather tame time-dependence. An *autonomous* equation $(\varDelta )$ is 1-periodic.

A nonautonomous set ${\mathcal {A}}\subseteq {\mathcal {U}}$ is called *positively* or *negatively invariant* (w.r.t. the difference equation $(\varDelta )$), if the respective inclusionholds; an *invariant* set ${\mathcal {A}}$ is both positively and negatively invariant, that is,  for all $t\in {\mathbb {I}}'$. One denotes ${\mathcal {A}}$ as $\theta$-*periodic*, if ${\mathcal {A}}(t)={\mathcal {A}}(t+\theta )$ holds for all $t\in {\mathbb {I}}$ with $t+\theta \in {\mathbb {I}}$.

The next two subsections provide some preparations on nonautonomous difference equations in Banach spaces $(X,\left\| \cdot \right\| )$:

### Semilinear Difference Equations

Let , $t\in {\mathbb {I}}'$, be a sequence of bounded linear operators. For a *linear difference equation*we define the *transition operator* $\varPhi :\left\{ (t,\tau )\in {\mathbb {I}}^2:\,\tau \le t\right\} \rightarrow L(X)$ byThen $(\varDelta )$ is understood as *semilinear*, if its right-hand side can be represented as2.5with continuous mappings , $t\in {\mathbb {I}}'$. The variation of constants formula [20, p. 100, Thm. 3.1.16] yields the general solution of $(\varDelta )$ in the form2.6The following result will be helpful in the construction of absorbing sets:

#### Lemma 2.1

Let  be of semilinear form (2.5) and suppose there exist reals $\alpha _t\ge 0$, $K\ge 1$ with

2.7 $$\begin{aligned} \left\| \varPhi (t,s)\right\| \le K\prod _{r=s}^{t-1}\alpha _r\quad \text {for all }s\le t. \end{aligned}$$

If there exist reals $a_t\ge 0$, $b_t\ge 0$ such that the nonlinearity fulfills2.8then the general solution of $(\varDelta )$ satisfies the estimate

2.9 $$\begin{aligned} \left\| \varphi (t;\tau ,u_\tau )\right\| \le K\left\| u_\tau \right\| \prod _{r=\tau }^{t-1}(\alpha _r+Ka_r)+K\sum _{s=\tau }^{t-1}b_s\prod _{r=s+1}^{t-1}(\alpha _r+Ka_r) \end{aligned}$$

for all $\tau \le t$ and $u_\tau \in U_\tau$.

#### Remark 2.2

(Linear growth) In case  on ${\mathbb {I}}'$ one can choose $K=1$, $\alpha _t=0$ in (2.7) and the estimate (2.9) simplifies to

$$\begin{aligned} \left\| \varphi (t;\tau ,u_\tau )\right\| \le \left\| u_\tau \right\| \prod _{r=\tau }^{t-1}a_r+\sum _{s=\tau }^{t-1}b_s\prod _{r=s+1}^{t-1}a_r \quad \text {for all }\tau \le t,\,u_\tau \in U_\tau . \end{aligned}$$

#### Proof

Let $\tau \in {\mathbb {I}}$. It is convenient to abbreviate $e_\alpha (t,s){:=}\prod _{r=s}^{t-1}\alpha _r$ and we first assume that $\alpha _t\ne 0$, $t\in {\mathbb {I}}'$. Given $u_\tau \in U_\tau$, from (2.6) and (2.7) we obtainand therefore the sequence $u(t){:=}\left\| \varphi (t;\tau ,u_\tau )\right\| e_\alpha (\tau ,t)$ satisfies

$$\begin{aligned} u(t) \le K\left\| u_\tau \right\| + K\sum _{s=\tau }^{t-1}b_se_\alpha (\tau ,s+1) + K\sum _{s=\tau }^{t-1}\frac{a_s}{\alpha _s}u(s)\quad \text {for all }\tau \le t. \end{aligned}$$

Thus, the Grönwall inequality from [20, p. 348, Prop. A.2.1(a)] implies

$$\begin{aligned} u(t) \le Ke_{1+\frac{Ka}{\alpha }}(t,\tau )\left\| u_\tau \right\| + K\sum _{s=\tau }^{t-1}b_se_\alpha (\tau ,s+1)e_{1+\frac{Ka}{\alpha }}(t,s+1) \end{aligned}$$

and consequently

$$\begin{aligned} \left\| \varphi (t;\tau ,u_\tau )\right\| \le Ke_{\alpha +Ka}(t,\tau )\left\| u_\tau \right\| +K\sum _{s=\tau }^{t-1}b_se_{\alpha +Ka}(t,s+1) \quad \text {for all }\tau \le t, \end{aligned}$$

which is the claimed inequality (2.9). $\square$

### Additive Difference Equations

We now address right-hand sides2.10where  is bounded and continuous, while , $t\in {\mathbb {I}}'$, is assumed to be completely continuous.

#### Lemma 2.3

If  is of additive form (2.10), then the general solution of $(\varDelta )$ satisfies

#### Proof

Since  for every $t\in {\mathbb {I}}'$ is continuous and bounded, their composition (2.2) is also continuous and bounded. The estimate for the Darbo constant of $\varphi (t;\tau ,\cdot )$ will be established by mathematical induction. For $t=\tau$ the assertion is clear, since $\varphi (\tau ;\tau ,\cdot )=I_X$ and the Lipschitz constant of the identity mapping is 1; it provides an upper bound for the Darbo constant (see [19, p. 81, Prop. 5.3]). For times $t\ge \tau$, from , which holds because  is completely continuous (cf. [19, p. 82, Prop. 5.4]), it follows thatfrom [19, pp. 79–80, Prop. 5.1]. This establishes the claim. $\square$

## Pullback Convergence

In this section, suppose that ${\mathbb {I}}$ is unbounded below and that , $t\in {\mathbb {I}}'$, i.e., $(\varDelta )$ generates a process on ${\mathcal {U}}$.

A difference equation $(\varDelta )$ is said to be *pullback asymptotically compact*, if for every $\tau \in {\mathbb {I}}$, every sequence $(s_n)_{n\in {\mathbb {N}}}$ in ${\mathbb {N}}_0$ with $\lim _{n\rightarrow \infty }s_n=\infty$ and every bounded sequence $(a_n)_{n\in {\mathbb {N}}}$ with $a_n\in {\mathcal {U}}(\tau -s_n)$, the sequence $\bigl (\varphi (\tau ;\tau -s_n,a_n)\bigr )_{n\in {\mathbb {N}}}$ possesses a convergent subsequence.

### Pullback Limit Sets

The *pullback limit set* $\omega _{{\mathcal {A}}}\subseteq {\mathcal {U}}$ of a bounded subset ${\mathcal {A}}\subseteq {\mathcal {U}}$ is given by the fibres

3.1 $$\begin{aligned} \omega _{{\mathcal {A}}}(\tau ){:=}\bigcap _{0\le s}\overline{\bigcup _{s\le t}\varphi (\tau ;\tau -t,{\mathcal {A}}(\tau -t))} \quad \text {for all }\tau \in {\mathbb {I}}. \end{aligned}$$

For pullback asymptotically compact nonautonomous difference equations $(\varDelta )$ it is shown in [20, p. 14, Thm. 1.2.25] that $\omega _{{\mathcal {A}}}$ is nonempty, compact, invariant and *pullback attracts* ${\mathcal {A}}$, i.e., the limit relation

3.2 $$\begin{aligned} \lim _{s\rightarrow \infty }{\text {dist}}\bigl (\varphi (\tau ;\tau -s,{\mathcal {A}}(\tau -s)),\omega _{{\mathcal {A}}}(\tau )\bigr )=0\quad \text {for all }\tau \in {\mathbb {I}} \end{aligned}$$

holds. For positively invariant sets ${\mathcal {A}}$ the defining relation (3.1) simplifies to

3.3 $$\begin{aligned} \omega _{{\mathcal {A}}}(\tau )=\bigcap _{0\le s}\overline{\varphi (\tau ;\tau -s,{\mathcal {A}}(\tau -s))}. \end{aligned}$$

Therefore, as a fundamental tool for the construction of pullback limit sets and attractors, as well as for forward attractors in Sect. 4.3, we state

#### Proposition 3.1

Suppose that $(\varDelta )$ has a nonempty, positively invariant, closed and bounded subset ${\mathcal {A}}\subseteq {\mathcal {U}}$. If $(\varDelta )$ is pullback asymptotically compact, then the fibres

3.4 $$\begin{aligned} {\mathcal {A}}^\star (\tau ){:=}\bigcap _{0\le s}\varphi (\tau ;\tau -s,{\mathcal {A}}(\tau -s)) \quad \text {for all }\tau \in {\mathbb {I}} \end{aligned}$$

define a maximal invariant, nonempty and compact nonautonomous set ${\mathcal {A}}^\star \subseteq {\mathcal {A}}$, which pullback attracts ${\mathcal {A}}$.

If the nonautonomous set ${\mathcal {A}}$ is even compact, then Proposition 3.1 applies without the asymptotic compactness assumption.

#### Proof

Since $(\varDelta )$ generates a continuous process $\varphi$ in discrete time, the assertion results via an adaption of [13, Prop. 5], where pullback asymptotic compactness yields that the intersection of the nested sets in (3.4) is nonempty. $\square$

### Pullback Attractors

A *pullback attractor* ${\mathcal {A}}^*\subseteq {\mathcal {U}}$ of $(\varDelta )$ is a nonempty, compact, invariant nonautonomous set which pullback attracts all bounded nonautonomous sets ${\mathcal {B}}\subseteq {\mathcal {U}}$. Bounded pullback attractors are unique and allow the dynamical characterisation

$$\begin{aligned} {\mathcal {A}}^*= \left\{ (\tau ,u)\in {\mathcal {U}}\, \left| \begin{array}{l} \text {there exists a bounded entire solution}\\ (\phi _t)_{t\in {\mathbb {I}}}\text { of }(\varDelta )\text { satisfying }\phi _\tau =u \end{array} \right. \right\} \end{aligned}$$

(cf. [20, p. 17, Cor. 1.3.4]). Despite being pullback attracting nonautonomous sets within ${\mathcal {A}}$, the set ${\mathcal {A}}^\star$ constructed in Proposition 3.1 needs not to be a pullback attractor, since nothing was assumed outside of ${\mathcal {A}}$. Remedy provides the notion of a *pullback dissipative* difference equation $(\varDelta )$. This means there exists a bounded set ${\mathcal {A}}\subseteq {\mathcal {U}}$ such that for every $\tau \in {\mathbb {I}}$ and every bounded nonautonomous set ${\mathcal {B}}\subseteq {\mathcal {U}}$ there is an *absorption time* $S=S(\tau ,{\mathcal {B}})\in {\mathbb {N}}$ such that

$$\begin{aligned} \varphi (\tau ;\tau -s,{\mathcal {B}}(\tau -s))\subseteq {\mathcal {A}}(\tau )\quad \text {for all }s\ge S. \end{aligned}$$

For a *uniformly pullback dissipative* equation $(\varDelta )$ the absorption time *S* is independent of $\tau$. One denotes ${\mathcal {A}}$ as a *pullback absorbing set*.

If ${\mathcal {A}}$ is pullback absorbing, then the set ${\mathcal {A}}^\star$ obtained from Proposition 3.1 becomes a pullback attractor, i.e., ${\mathcal {A}}^\star ={\mathcal {A}}^*$, and one has the characterisation

3.5 $$\begin{aligned} {\mathcal {A}}^*=\omega _{{\mathcal {A}}}. \end{aligned}$$

A possibility to construct pullback absorbing sets provides

#### Proposition 3.2

(Pullback absorbing set) On a Banach space *X*, let $\rho >0$ and  be of semilinear form (2.5) satisfying (2.7), (2.8). If the limit relations

$$\begin{aligned} \lim _{s\rightarrow \infty }\prod _{r=\tau -s}^{\tau -1}(\alpha _r+Ka_r)&=0,&R_\tau&{:=}K\sum _{s=-\infty }^{\tau -1}b_s\prod _{r=s+1}^{\tau -1}(\alpha _r+Ka_r)<\infty \end{aligned}$$

hold for all $\tau \in {\mathbb {I}}$, then the difference equation $(\varDelta )$ is pullback dissipative with absorbing set ${\mathcal {A}}{:=}\left\{ (\tau ,u)\in {\mathcal {U}}:\,\left\| u\right\| \le \rho +R_\tau \right\}$. In case $\lim _{s\rightarrow \infty }\sup _{\tau \in {\mathbb {I}}}\prod _{r=\tau -s}^{\tau -1}(\alpha _r+Ka_r)=0$ holds, the difference equation $(\varDelta )$ is uniformly pullback dissipative.

#### Proof

The assertion follows from Lemma 2.1 by passing over to the pullback limit $\tau \rightarrow -\infty$ in the estimate (2.9). $\square$

A construction of pullback attractors ${\mathcal {A}}^*$ based on set contractions, rather than asymptotic compactness, is suitable for later applications to integrodifference equations (see Sect. 5):

#### Theorem 3.3

If a difference equation $(\varDelta )$ of additive form (2.10) is uniformly pullback dissipative andholds, then there exists a unique bounded pullback attractor of $(\varDelta )$.

#### Remark 3.4

(Periodic equations) For $\theta$-periodic difference equations $(\varDelta )$ and sets ${\mathcal {A}}$, it results from (2.4) that also the pullback limit sets $\omega _{\mathcal {A}}$ from (3.1), the set ${\mathcal {A}}^\star$ from Proposition 3.1 and the pullback attractor ${\mathcal {A}}^*$ are $\theta$-periodic (cf. [20, pp. 21ff, Sect. 1.4]). Furthermore, Theorem 3.3 applies when .

#### Proof

The terminology of [20] and results therein will be used. Let $\hat{\mathcal {B}}$ denote the family of all bounded sets in ${\mathbb {I}}\times X$. Then Lemma 2.3 ensures that the general solution $\varphi (t;\tau ,\cdot )$ is $\hat{\mathcal {B}}$-contracting in the sense of [20, p. 15, Def. 1.2.26(i)].

Since $(\varDelta )$ has a bounded absorbing set ${\mathcal {A}}$, for every bounded nonautonomous set ${\mathcal {B}}$, there exists an $S\in {\mathbb {N}}_0$ such that $\varphi (\tau ;\tau -s,{\mathcal {B}}(\tau -s))\subseteq {\mathcal {A}}(t)$ holds for all $s\ge S$. This implies that the *S*-truncated orbit $\gamma _{\mathcal {B}}^S$, fibrewise given by

$$\begin{aligned} \gamma _{\mathcal {B}}^S(\tau ) {:=} \bigcup _{s\ge S}\varphi (\tau ;\tau -s,{\mathcal {B}}(\tau -s))\subseteq {\mathcal {A}}(\tau )\quad \text {for all }\tau \in {\mathbb {I}}, \end{aligned}$$

is bounded. Hence, [20, p. 16, Prop. 1.2.30] implies that $(\varDelta )$ is $\hat{\mathcal {B}}$-asymptotically compact, so $(\varDelta )$ has a pullback attractor ${\mathcal {A}}^*$ by [20, p. 19, Thm. 1.3.9].

Finally, the pullback attractor ${\mathcal {A}}^*$ is contained in the closure of the absorbing set ${\mathcal {A}}$, so is bounded and thus uniquely determined. $\square$

## Forward Convergence

In the previous section, we constructed pullback attractors of pullback asymptotically compact nonautonomous difference equations $(\varDelta )$ as pullback limit sets of such absorbing sets. Our next aim is to provide related notions in forward time. Due to the conceptional difference between pullback and forward convergence some modifications are necessary, yet.

Above all, this requires a discrete interval ${\mathbb {I}}$ to be unbounded above. Now the right-hand sides , $t\in {\mathbb {I}}$, are defined on a common closed subset $U_t=U\subseteq X$, i.e., the extended state space ${\mathcal {U}}={\mathbb {I}}\times U$ has constant fibres. Therefore, the general solution $\varphi :\left\{ (t,\tau ,u)\in {\mathbb {I}}\times {\mathcal {U}}:\,\tau \le t\right\} \rightarrow U$ is well-defined.

Given a nonautonomous set ${\mathcal {A}}\subseteq {\mathcal {U}}$, a difference equation $(\varDelta )$ is said to be

- ${\mathcal {A}}$*-asymptotically compact*, if there exists a compact set $K\subseteq U$ such that *K* forward attracts ${\mathcal {A}}(\tau )$, i.e.,
  $$\begin{aligned} \lim _{s\rightarrow \infty }{\text {dist}}\bigl (\varphi (\tau +s;\tau ,{\mathcal {A}}(\tau )),K\bigr )=0 \quad \text {for all }\tau \in {\mathbb {I}}, \end{aligned}$$
- *strongly* ${\mathcal {A}}$*-asymptotically compact*, if there exists a compact set $K\subseteq U$ so that every sequence $\bigl ((s_n,\tau _n)\bigr )_{n\in {\mathbb {N}}}$ in ${\mathbb {N}}\times {\mathbb {I}}$ with $s_n\rightarrow \infty$, $\tau _n\rightarrow \infty$ as $n\rightarrow \infty$ yields
  $$\begin{aligned} \lim _{n\rightarrow \infty }{\text {dist}}\bigl (\varphi (\tau _n+s_n;\tau _n,{\mathcal {A}}(\tau _n)), K\bigr )=0. \end{aligned}$$

### Remark 4.1

If ${\mathcal {A}}$ is positively invariant, then strong ${\mathcal {A}}$-asymptotic compactness (needed in Theorem 4.10 below) is a tightening of ${\mathcal {A}}$-asymptotic compactness (required in Theorem 4.9). Indeed, suppose that the sequence $({\text {dist}}\bigl (\varphi (t_n;\tau ,{\mathcal {A}}(\tau )),K\bigr ))_{n\in {\mathbb {N}}}$ does not converge to 0. Hence, the strong ${\mathcal {A}}$-asymptotic compactness of $(\varDelta )$ and positive invariance of ${\mathcal {A}}$ yields the contradiction

### Forward Limit Sets

Let us investigate the forward dynamics of $(\varDelta )$ inside a nonautonomous set ${\mathcal {A}}$. We first capture the forward limit points from a single fibre ${\mathcal {A}}(\tau )$:

#### Lemma 4.2

Suppose that ${\mathcal {A}}\ne \emptyset$ is a bounded nonautonomous set. If $(\varDelta )$ is ${\mathcal {A}}$-asymptotically compact with a compact subset $K\subseteq U$, then the fibres

4.1 $$\begin{aligned} \varOmega _{\mathcal {A}}(\tau ){:=}\bigcap _{0\le s}\overline{\bigcup _{s\le t}\varphi (\tau +t;\tau ,{\mathcal {A}}(\tau ))}\subseteq K \quad \text {for all }\tau \in {\mathbb {I}} \end{aligned}$$

are nonempty, compact, and forward attract ${\mathcal {A}}(\tau )$, i.e.,

4.2 $$\begin{aligned} \lim _{s\rightarrow \infty }{\text {dist}}\bigl (\varphi (\tau +s;\tau ,{\mathcal {A}}(\tau )),\varOmega _{\mathcal {A}}(\tau )\bigr )=0. \end{aligned}$$

An analogous result for pullback limit sets is given in [20, p. 9, Lemma 1.2.12].

#### Remark 4.3

(Characterisation of $\varOmega _{{\mathcal {A}}}(\tau )$) The fibres $\varOmega _{\mathcal {A}}(\tau )$, $\tau \in {\mathbb {I}}$, consist of points *v* such that there is a sequence $\bigl ((s_n,a_n)\bigr )_{n\in {\mathbb {N}}}$ with $\lim _{n\rightarrow \infty }s_n=\infty$, $a_n\in {\mathcal {A}}(\tau )$ and

4.3 $$\begin{aligned} \lim _{n\rightarrow \infty }\varphi (\tau +s_n;\tau ,a_n)=v. \end{aligned}$$

This readily yields the monotonicity ${\mathcal {A}}_1\subseteq {\mathcal {A}}_2$ $\Rightarrow$ $\varOmega _{{\mathcal {A}}_1}(\tau )\subseteq \varOmega _{{\mathcal {A}}_2}(\tau )$ for all $\tau \in {\mathbb {I}}$.

#### Proof

Let $\tau \in {\mathbb {I}}$. Given a sequence $y_n{:=}\varphi (\tau +s_n;\tau ,a_n)\in \varphi (\tau +s_n;\tau ,{\mathcal {A}}(\tau ))$ with $s_n\xrightarrow [n\rightarrow \infty ]{}\infty$ and $a_n\in {\mathcal {A}}(\tau )$, by the ${\mathcal {A}}$-asymptotic compactness of $(\varDelta )$, we obtain

$$\begin{aligned} 0\le {\text {dist}}\bigl (y_n,K\bigr )\le {\text {dist}}\bigl (\varphi (\tau +s_n,\tau ,{\mathcal {A}}(\tau )),K\bigr )\xrightarrow [n\rightarrow \infty ]{}0. \end{aligned}$$

Since $K\subseteq U$ is compact, ${\text {dist}}\bigl (y_n,K\bigr )=\min _{k\in K}d(y_n,k)$. This implies that there exist a sequence $(k_n)_{n\in {\mathbb {N}}}$ in *K* satisfying

$$\begin{aligned} d(y_n,k_n) = {\text {dist}}\bigl (y_n,K\bigr )\xrightarrow [n\rightarrow \infty ]{}0, \end{aligned}$$

and a subsequence $(k_{n_j})_{j\in {\mathbb {N}}}$ converging to $\bar{k}\in K$. Thus,

$$\begin{aligned} 0\le d\bigl (y_{n_j},\bar{k}\bigr ) \le d\bigl (y_{n_j},k_{n_j}\bigr )+d\bigl (k_{n_j},\bar{k}\bigr ) ={\text {dist}}\bigl (y_{n_j},K\bigr )+d\bigl (k_{n_j},\bar{k}\bigr )\xrightarrow [j\rightarrow \infty ]{}0, \end{aligned}$$

which implies that the subsequence $(y_{n_j})_{j\in {\mathbb {N}}}$ converges to $\bar{k}$. Hence, by the characterisation (4.3), $\bar{k}\in \varOmega _{\mathcal {A}}(\tau )$, i.e., $\varOmega _{\mathcal {A}}(\tau )$ is nonempty.

Now choose a sequence $(v_n)_{n\in {\mathbb {N}}}$ in $\varOmega _{\mathcal {A}}(\tau )$. By Remark 4.3, for each fixed $n\in {\mathbb {N}}$, there is a sequence $\bigl ((s_m^n,a_m^n)\bigr )_{m\in {\mathbb {N}}}$ satisfying $\lim _{m\rightarrow \infty }s_m^n=\infty$ and $a_m^n\in {\mathcal {A}}(\tau )$ for all $m\in {\mathbb {N}}$ such that

$$\begin{aligned} \lim _{m\rightarrow \infty }\varphi (\tau +s_m^n;\tau ,a_m^n)=v_n, \end{aligned}$$

i.e., for every $\varepsilon >0$, there is a $M=M(n,\varepsilon )\in {\mathbb {N}}$ such that

$$\begin{aligned} d\bigl (\varphi (\tau +s_m^n;\tau ,a_m^n),v_n\bigr )<\varepsilon \quad \text {for all }m\ge M. \end{aligned}$$

Since $\lim _{m\rightarrow \infty }s_m^n=\infty$, there is a $M'=M'(n)\in {\mathbb {N}}$ satisfying $s_m^n>n$ for all $m\ge M'$. Pick $\varepsilon =\frac{1}{n}$, $(\bar{s}_n)_{n\in {\mathbb {N}}}$ as a subsequence $(s_{m_n}^n)_{n\in {\mathbb {N}}}$ of $(s_m^n)_{n\in {\mathbb {N}}}$ and $(\bar{a}_n)_{n\in {\mathbb {N}}}$ as a subsequence $(a_{m_n}^n)_{n\in {\mathbb {N}}}$ of $(a_m^n)_{n\in {\mathbb {N}}}$ in such a way that

$$\begin{aligned} m_1&=\max \left\{ 1,M,M'\right\} +1,&m_{n+1}&=\max \left\{ 1,M,M',m_n\right\} +1 \quad \text {for all }n\in {\mathbb {N}}. \end{aligned}$$

Clearly, $m_{n+1}>\max \left\{ 1,M,M',m_n\right\}$ for $n\in {\mathbb {N}}$. Hence, we constructed a sequence $\bigl ((\bar{s}_n,\bar{a}_n)\bigr )_{n\in {\mathbb {N}}}$ with $\lim _{n\rightarrow \infty }\bar{s}_n=\infty$ and $\bar{a}_n\in {\mathcal {A}}(\tau )$ such that

$$\begin{aligned} d\bigl (\varphi (\tau +\bar{s}_n;\tau ,\bar{a}_n),v_n\bigr )<\tfrac{1}{n} \quad \text {for all }n\in {\mathbb {N}}. \end{aligned}$$

Therefore,

$$\begin{aligned} 0\le {\text {dist}}\bigl (v_n,K\bigr )&\le d\bigl (\varphi (\tau +\bar{s}_n;\tau ,\bar{a}_n),v_n\bigr )+{\text {dist}}\bigl (\varphi (\tau +\bar{s}_n;\tau ,{\mathcal {A}}(\tau )),K\bigr )\\&\le \tfrac{1}{n}+{\text {dist}}\bigl (\varphi (\tau +\bar{s}_n;\tau ,{\mathcal {A}}(\tau )),K\bigr ) \xrightarrow [n\rightarrow \infty ]{}0. \end{aligned}$$

Similarly as above, because *K* is compact, there is a subsequence $(v_{n_j})_{j\in {\mathbb {N}}}$ converging to $\bar{v}\in K$. Moreover, since $\varOmega _{\mathcal {A}}(\tau )$ is closed by definition, $\bar{v}\in \varOmega _{\mathcal {A}}(\tau )$, which implies that $\varOmega _{\mathcal {A}}(\tau )$ is compact. Also note that $\lim _{n\rightarrow \infty }{\text {dist}}\bigl (v_n,K\bigr )=0$, so $v_n\in K$, i.e., $\varOmega _{\mathcal {A}}(\tau )\subseteq K$.

Suppose that $\varOmega _{\mathcal {A}}(\tilde{\tau })$ does not forward attract ${\mathcal {A}}(\tilde{\tau })$ for some $\tilde{\tau }\in {\mathbb {I}}$, i.e., there exist a real $\tilde{\varepsilon }>0$ and a sequence $(\tilde{s}_n)_{n\in {\mathbb {N}}}$ in ${\mathbb {N}}$ with $\lim _{n\rightarrow \infty }\tilde{s}_n=\infty$ and

4.4 $$\begin{aligned} {\text {dist}}\bigl (\varphi (\tilde{\tau }+\tilde{s}_n;\tilde{\tau },{\mathcal {A}}(\tilde{\tau })),\varOmega _{\mathcal {A}}(\tilde{\tau })\bigr )\ge \tilde{\varepsilon } \quad \text {for all }n\in {\mathbb {N}}. \end{aligned}$$

Although the supremum in the Hausdorff semidistance in the left-hand side of (4.4) may not be attained due to no condition ensuring that the image $\varphi (\tilde{\tau }+s_n;\tilde{\tau },{\mathcal {A}}(\tilde{\tau }))$ is compact, there still exists a point $\tilde{y}_n{:=}\varphi (\tilde{\tau }+\tilde{s}_n;\tilde{\tau },\tilde{a}_n) \in \varphi (\tilde{\tau }+\tilde{s}_n;\tilde{\tau },{\mathcal {A}}(\tilde{\tau }))$ for each $n\in {\mathbb {N}}$ with $\tilde{a}_n\in {\mathcal {A}}(\tilde{\tau })$ such that

$$\begin{aligned} {\text {dist}}\bigl (\varphi (\tilde{\tau }+\tilde{s}_n;\tilde{\tau },{\mathcal {A}}(\tilde{\tau })), \varOmega _{\mathcal {A}}(\tilde{\tau })\bigr )-\tfrac{\tilde{\varepsilon }}{2}&\le {\text {dist}}\bigl (\tilde{y}_n, \varOmega _{\mathcal {A}}(\tilde{\tau })\bigr )\\&\le {\text {dist}}\bigl (\varphi (\tilde{\tau }+\tilde{s}_n;\tilde{\tau },{\mathcal {A}}(\tilde{\tau })\bigr ), \varOmega _{\mathcal {A}}(\tilde{\tau })). \end{aligned}$$

The above inequalities in fact give ${\text {dist}}\bigl (\tilde{y}_n,\varOmega _{\mathcal {A}}(\tilde{\tau })\bigr )\ge \tfrac{\tilde{\varepsilon }}{2}$ for all $n\in {\mathbb {N}}$. On the other hand, since $\tilde{y}_n\in \varphi (\tilde{\tau }+s_n;\tilde{\tau },{\mathcal {A}}(\tilde{\tau }))$, we obtain

$$\begin{aligned} {\text {dist}}\bigl (\tilde{y}_n,K\bigr )\le {\text {dist}}\bigl (\varphi (\tilde{\tau }+\tilde{s}_n;\tilde{\tau },{\mathcal {A}}(\tilde{\tau })),K\bigr )\xrightarrow [n\rightarrow \infty ]{}0 \end{aligned}$$

and thus $\tilde{y}_n\in K$. Moreover, since *K* is compact, there is a convergent subsequence $(\tilde{y}_{n_j})_{j\in {\mathbb {N}}}$ with limit $\tilde{y}\in K$. This shows $\tilde{y}\in \varOmega _{\mathcal {A}}(\tilde{\tau })$ by definition, and thus

$$\begin{aligned} {\text {dist}}\bigl (\tilde{y},\varOmega _{\mathcal {A}}(\tilde{\tau })\bigr )<\varepsilon \quad \text {for all }\varepsilon >0, \end{aligned}$$

a contradiction to (4.4). Hence, every $\varOmega _{\mathcal {A}}(\tau )$ must forward attract ${\mathcal {A}}(\tau )$. $\square$

#### Corollary 4.4

If in addition ${\mathcal {A}}$ is positively invariant w.r.t. $(\varDelta )$, then the inclusions $\varOmega _{\mathcal {A}}(\tau )\subseteq \varOmega _{\mathcal {A}}(\tau +1)$ hold for all $\tau \in {\mathbb {I}}$.

Owing to the positive invariance of ${\mathcal {A}}$, every $\varOmega _{\mathcal {A}}(\tau )$ can also be written as

4.5 $$\begin{aligned} \varOmega _{\mathcal {A}}(\tau ) = \bigcap _{0\le s} \overline{\varphi (\tau +s;\tau ,{\mathcal {A}}(\tau ))}\quad \text {for all }\tau \in {\mathbb {I}}. \end{aligned}$$

Comparing the respective relations (4.1) and (3.1), (4.2) and (3.2), (4.5) and (3.3) shows that the fibres $\varOmega _{{\mathcal {A}}}(\tau )$ are counterparts to the pullback limit set $\omega _{{\mathcal {A}}}$. However, their invariance property is missing. This is easily demonstrated by

#### Example 4.5

Let ${\mathbb {I}}={\mathbb {N}}_0$ and $\alpha \in (0,\tfrac{1}{2}]$. The difference equation $u_{t+1}=\alpha u_t+\alpha ^t$ in ${\mathbb {R}}$ possesses the positively invariant and bounded set ${\mathcal {A}}={\mathbb {N}}_0\times [-\tfrac{1}{\alpha },\tfrac{1}{\alpha }]$. This yields the apparently not even positively invariant sets $\varOmega _{\mathcal {A}}(\tau )=\left\{ 0\right\}$ for all $\tau \in {\mathbb {N}}_0$.

#### Proof

Let $\tau \in {\mathbb {I}}$. Given a point $v\in \varOmega _{\mathcal {A}}(\tau )$, thanks to  and the process property (2.3), we obtain

$$\begin{aligned} v\in \varphi (\tau +s;\tau ,{\mathcal {A}}(\tau ))\subseteq \varphi (\tau +s;\tau +1,{\mathcal {A}}(\tau +1))\subseteq U \quad \text {for all }s>0. \end{aligned}$$

This implies $$\varOmega _{\mathcal {A}}(\tau )\subseteq \varOmega _{\mathcal {A}}(\tau +1)\subseteq U$$. $$\square $$

While the fibres $\varOmega _{{\mathcal {A}}}(\tau )$ from Lemma 4.2 yield the long term behaviour starting from a single fibre of ${\mathcal {A}}$, the following result addresses all forward limit sets of $(\varDelta )$ originating from within an entire nonautonomous set ${\mathcal {A}}$.

#### Theorem 4.6

(Forward $\omega$-limit sets) Suppose that ${\mathcal {A}}\ne \emptyset$ is positively invariant and bounded. If $(\varDelta )$ is ${\mathcal {A}}$-asymptotically compact with a compact subset $K\subseteq U$, then

$$\begin{aligned} \omega _{{\mathcal {A}}}^-&{:=}\bigcap _{\tau \in {\mathbb {I}}}\varOmega _{\mathcal {A}}(\tau ),&\omega _{\mathcal {A}}^+&{:=}\overline{\bigcup _{\tau \in {\mathbb {I}}}\varOmega _{\mathcal {A}}(\tau )}\subseteq K \end{aligned}$$

are nonempty and compact. In particular, $\omega _{{\mathcal {A}}}^+$ forward attracts ${\mathcal {A}}$, i.e.,

$$\begin{aligned} \lim _{s\rightarrow \infty }{\text {dist}}\bigl (\varphi (\tau +s;\tau ,{\mathcal {A}}(\tau )),\omega _{{\mathcal {A}}}^+\bigr )&=0 \quad \text {for all }\tau \in {\mathbb {I}}\end{aligned}$$

and called *forward* $\omega$*-limit set* of ${\mathcal {A}}$.

Due to Corollary 4.4, $\omega _{{\mathcal {A}}}^+$ is a union over nondecreasing sets and actually a limit.

#### Remark 4.7

(Characterisation of $\omega _{{\mathcal {A}}}^+$) The forward $\omega$-limit set $\omega _{\mathcal {A}}^+$ consists of all points *v* such that there is a sequence $\bigl ((s_n,\tau _n,a_n)\bigr )_{n\in {\mathbb {N}}}$ with $\lim _{n\rightarrow \infty }\tau _n=\infty$, $(\tau _n,a_n)\in {\mathcal {A}}$ and $s_n\in {\mathbb {N}}_0$ satisfying

$$\begin{aligned} \lim _{n\rightarrow \infty }\varphi (\tau _n+s_n;\tau _n,a_n)=v. \end{aligned}$$

#### Remark 4.8

(Periodic equations) For $\theta$-periodic difference equations $(\varDelta )$ and sets ${\mathcal {A}}$ the fibres $\varOmega _{\mathcal {A}}(\tau )$ are $\theta$-periodic due to (2.4) and (4.1). If ${\mathcal {A}}$ is moreover positively invariant, then $\varOmega _{\mathcal {A}}$ are even constant and thus $\omega _{\mathcal {A}}^-=\omega _{\mathcal {A}}^+=\varOmega _{\mathcal {A}}(\tau )$ for all $\tau \in {\mathbb {Z}}$.

#### Proof of Theorem 4.6

Since $\varOmega _{\mathcal {A}}(\tau )$ is nonempty, there exists a point $v\in \varOmega _{\mathcal {A}}(\tau )$ for all $\tau \in {\mathbb {I}}$. This implies that *v* is also contained in $\omega _{\mathcal {A}}^+$, i.e., the forward $\omega$-limit set $\omega _{\mathcal {A}}^+$ is nonempty. From Lemma 4.2, we know that $\varOmega _{\mathcal {A}}(\tau )\subseteq K$ for each $\tau \in {\mathbb {I}}$. This yields $\omega _{\mathcal {A}}^+\subseteq K$. Moreover, since *K* is compact and $\omega _{\mathcal {A}}^+$ is closed, $\omega _{\mathcal {A}}^+$ is also compact. The claimed limit relation is a consequence of (4.2) and $\varOmega _{\mathcal {A}}(\tau )\subseteq \omega _{{\mathcal {A}}}^+$.

The properties of $\omega _{{\mathcal {A}}}^-$ are an immediate consequence of the fact that $\omega _{{\mathcal {A}}}^-$ is an intersection of nested compact sets (cf. [23, p. 23, Lemma 22.2(5)]). $\square$

Note that Example 4.5 demonstrates that both the set $\omega _{\mathcal {A}}^-$, as well as the forward limit sets $\omega _{\mathcal {A}}^+$ constructed in Theorem 4.6 are not invariant or even positively invariant. Yet, under additional assumptions weaker forms of invariance hold:

#### Theorem 4.9

(Asymptotic positive invariance) Suppose that $(\varDelta )$ is ${\mathcal {A}}$-asymptotically compact with a compact subset $K\subseteq U$ for a bounded, positively invariant ${\mathcal {A}}\ne 0$. If for every sequence $\bigl ((s_n,\tau _n)\bigr )_{n\in {\mathbb {N}}}$ in ${\mathbb {N}}_0\times {\mathbb {I}}$ with $\lim _{n\rightarrow \infty }\tau _n=\infty$, one has

$$\begin{aligned} \lim _{n\rightarrow \infty }{\text {dist}}\bigl (\varphi (\tau _n+s_n;\tau _n,K),K\bigr )=0, \end{aligned}$$

then the forward $\omega$-limit set $\omega _{\mathcal {A}}^+$ is *asymptotically positively invariant*, that is, for every strictly decreasing sequence $\varepsilon _n\searrow 0$, there exists a strictly increasing sequence $T_n\nearrow \infty$ in ${\mathbb {I}}$ as $n\rightarrow \infty$ such that

4.6 $$\begin{aligned} \varphi (\tau +s;\tau ,\omega _{\mathcal {A}}^+)\subseteq B_{\varepsilon _n}\bigl (\omega _{\mathcal {A}}^+\bigr )\quad \text {for all }T_n\le \tau ,\,s\in {\mathbb {N}}_0. \end{aligned}$$

Recall the definition of the neighborhoods $B_{\varepsilon _n}\bigl (\omega _{\mathcal {A}}^+\bigr )$ and thus (4.6) reads as

$$\begin{aligned} {\text {dist}}\bigl (\varphi (\tau +s;\tau ,\omega _{\mathcal {A}}^+),\omega _{\mathcal {A}}^+\bigr )<\varepsilon _n \quad \text {for all }T_n\le \tau ,\,s\in {\mathbb {N}}_0. \end{aligned}$$

#### Proof

Suppose by contradiction that there exists a fixed $\varepsilon _1>0$ so that there is a sequence $\bigl ((s_n^1,\tau _n)\bigr )_{n\in {\mathbb {N}}}$ with $0\le s_n^1=s_n^1(\varepsilon _1)\le T_0(\tau _n,\varepsilon _1)$ and $\tau _n\rightarrow \infty$ as $n\rightarrow \infty$ satisfying

4.7 $$\begin{aligned} {\text {dist}}\bigl (\varphi (\tau _n+s_n^1;\tau _n,\omega _{\mathcal {A}}^+),\omega _{\mathcal {A}}^+\bigr )\ge \varepsilon _1 \quad \text {for all }n\in {\mathbb {N}}. \end{aligned}$$

Since $\varphi$ is continuous and $\omega _{\mathcal {A}}^+$ is compact due to Theorem 4.6, $\varphi (\tau _n+s_n^1;\tau _n,\omega _{\mathcal {A}}^+)$ is also a compact set. This implies that there exists a

$$\begin{aligned} y_n^1=y_n^1(\varepsilon _1){:=}\varphi (\tau _n+s_n^1;\tau _n,w_n^1) \in \varphi (\tau _n+s_n^1;\tau _n,\omega _{\mathcal {A}}^+)\subseteq \varphi (\tau _n+s_n^1;\tau _n,K) \end{aligned}$$

with $w_n^1=w_n^1(\varepsilon _1)\in \omega _{\mathcal {A}}^+\subseteq K$ such that

$$\begin{aligned} {\text {dist}}\bigl (y_n^1,\omega _{\mathcal {A}}^+\bigr )&={\text {dist}}\bigl (\varphi (\tau _n+s_n^1;\tau _n,w_n^1),\omega _{\mathcal {A}}^+\bigr )\\&={\text {dist}}\bigl (\varphi (\tau _n+s_n^1;\tau _n,\omega _{\mathcal {A}}^+),\omega _{\mathcal {A}}^+\bigr )\ge \varepsilon _1 \quad \text {for all }n\in {\mathbb {N}}. \end{aligned}$$

On the other hand, with $y_n^1\in \varphi (\tau _n+s_n^1;\tau _n,K)$, by the assumption, we obtain

$$\begin{aligned} 0\le {\text {dist}}\bigl (y_n^1,K\bigr )\le {\text {dist}}\bigl (\varphi (\tau _n+s_n^1;\tau _n,K),K\bigr )\xrightarrow [n\rightarrow \infty ]{}0, \end{aligned}$$

implying that $y_n^1\in K$. Additionally, since the set *K* is compact, there is a subsequence $(y_{n_j}(\varepsilon _1))_{j\in {\mathbb {N}}}$ converging to $\bar{y}_1=\bar{y}_1(\varepsilon _1)\in K$. Therefore, by definition, the inclusion $\bar{y}_1\in \varOmega _{\mathcal {A}}(\tau )\subseteq \omega _{\mathcal {A}}^+$ leads to $\bar{y}_1\in \omega _{\mathcal {A}}^+$, i.e., ${\text {dist}}\bigl (\bar{y}_1,\omega _{\mathcal {A}}^+\bigr )<\varepsilon$ for all $\varepsilon >0$, a contradiction to (4.7). Thus, for this $\varepsilon _1>0$, there exists an integer $s_1=s_1(\varepsilon _1)$ large enough such that

$$\begin{aligned} {\text {dist}}\bigl (\varphi (\tau +s_1;\tau ,\omega _{\mathcal {A}}^+),\omega _{\mathcal {A}}^+\bigr )<\varepsilon _1. \end{aligned}$$

Repeating inductively with $\varepsilon _{n+1}<\varepsilon _n$ and $s_{n+1}(\varepsilon _{n+1})>s_n(\varepsilon _n)$ for all $n\in {\mathbb {N}}$, we then obtain that $\omega _{\mathcal {A}}^+$ is asymptotically positively invariant. $\square$

#### Theorem 4.10

(Asymptotic negative invariance) Suppose that $(\varDelta )$ is strongly ${\mathcal {A}}$-asymptotically compact with a compact subset $K\subseteq U$ for a bounded, positively invariant ${\mathcal {A}}\ne 0$. If for every $\varepsilon >0$ and $T\in {\mathbb {N}}$, there exists a real $\delta =\delta (\varepsilon ,T)>0$ such that for all $\tau \in {\mathbb {I}}$, one has the implication

4.8 $$\begin{aligned} \left. \begin{array}{c} u_0,v_0\in {\mathcal {A}}(\tau )\cup K,\\ d(u_0,v_0)<\delta \end{array} \right\} \Rightarrow \sup _{0\le s\le T} d\bigl (\varphi (\tau +s;\tau ,u_0),\varphi (\tau +s;\tau ,v_0)\bigr )<\varepsilon , \end{aligned}$$

then the forward $\omega$-limit set $\omega _{\mathcal {A}}^+$ is *asymptotically negatively invariant*, that is, for all $u\in \omega _{\mathcal {A}}^+$, $\varepsilon >0$ and $T\in {\mathbb {N}}$, there are integers $s^*=s^*(\varepsilon )$ satisfying $\tau +s^*-T\in {\mathbb {I}}$ and $u_\varepsilon ^*\in \omega _{\mathcal {A}}^+$ such that

$$\begin{aligned} d\bigl (\varphi (\tau +s^*;\tau +s^*-T,u_\varepsilon ^*),u\bigr )<\varepsilon . \end{aligned}$$

#### Proof

Consider reals $\varepsilon >0$ and $T\in {\mathbb {N}}$ and take a point $u\in \omega _{\mathcal {A}}^+$. Thanks to Remark 4.7, there is a sequence $\bigl ((s_n,\tau _n,a_n)\bigr )_{n\in {\mathbb {N}}}$ with $T<s_n=s_n(\varepsilon )\rightarrow \infty$, $\tau _n\rightarrow \infty$ as $n\rightarrow \infty$, $\tau _n\in {\mathbb {I}}$, $\tau _n+s_n-T\in {\mathbb {I}}$ and $a_n=a_n(\varepsilon )\in {\mathcal {A}}(\tau _n)$, and an integer $N=N_1(\varepsilon )$ with

$$\begin{aligned} d\bigl (\varphi (\tau _n+s_n;\tau _n,a_n),u\bigr )<\tfrac{\varepsilon }{2} \quad \text {for all }n\ge N_1(\varepsilon ). \end{aligned}$$

Given a sequence $y_n{:=}\varphi (t^*_n-T;\tau _n,a_n)\in \varphi (t^*_n-T;\tau _n,{\mathcal {A}}(\tau _n)) \subseteq {\mathcal {A}}(t^*_n-T)$ with $t^*_n=t^*_n(\varepsilon ){:=}\tau _n+s_n$, $s_n-T\rightarrow \infty$, $\tau _n\rightarrow \infty$ as $n\rightarrow \infty$, $\tau _n\in {\mathbb {I}}$ and $a_n\in {\mathcal {A}}(\tau _n)$, by the strong ${\mathcal {A}}$-asymptotic compactness of $(\varDelta )$, we obtain

$$\begin{aligned} 0\le {\text {dist}}\bigl (y_n,K\bigr )\le {\text {dist}}\bigl (\varphi (t^*_n-T;\tau _n,{\mathcal {A}}(\tau _n)), K\bigr )\xrightarrow [n\rightarrow \infty ]{}0. \end{aligned}$$

Since *K* is compact, ${\text {dist}}\bigl (y_n,K\bigr )=\min _{k\in K} d(y_n,k)$. This implies that there exist a sequence $(k_n)_{n\in {\mathbb {N}}}$ in *K* such that

$$\begin{aligned} d(y_n,k_n)={\text {dist}}\bigl (y_n,K\bigr )\xrightarrow [n\rightarrow \infty ]{}0, \end{aligned}$$

and a subsequence $(k_{n_j})_{j\in {\mathbb {N}}}$ converging to $\bar{k}=\bar{k}(\varepsilon )\in K$. Thus,

$$\begin{aligned} 0\le d\bigl (y_{n_j},\bar{k}\bigr ) \le d\bigl (y_{n_j},k_{n_j}\bigr )+d\bigl (k_{n_j},\bar{k}\bigr ) ={\text {dist}}\bigl (y_n,K\bigr )+d\bigl (k_{n_j},\bar{k}\bigr )\xrightarrow [n\rightarrow \infty ]{}0, \end{aligned}$$

implying $y_{n_j}{:}{=}\varphi (t^*_{n_j}-T;\tau _{n_j},a_{n_j})\xrightarrow [j\rightarrow \infty ]{}\bar{k}$ with $t^*_{n_j}{:}{=}\tau _{n_j}+s_{n_j}$. Hence, by Remark 4.7, one has $\bar{k}\in \omega _{\mathcal {A}}^+$. Moreover, with $y_{n_j}\in {\mathcal {A}}(t^*_{n_j}-T)$ and $\bar{k}\in K$, by the assumption, we obtain for an integer $N_2(\varepsilon ,T)$ large enough,

$$\begin{aligned} d\bigl (\varphi (t^*_{n_j};t^*_{n_j}-T,y_{n_j}), \varphi (t^*_{n_j};t^*_{n_j}-T,\bar{k})\bigr )<\tfrac{\varepsilon }{2} \quad \text {for all }n_j\ge N_2(\varepsilon ,T). \end{aligned}$$

Now the triangle inequality and the process property (2.3) yield

$$\begin{aligned}&d\bigl (\varphi (t^*_{n_j};t^*_{n_j}-T,\bar{k}),u\bigr )\\&\quad \le d\bigl (\varphi (t^*_{n_j};t^*_{n_j}-T,\bar{k}), \varphi (t^*_{n_j};t^*_{n_j}-T,y_{n_j})\bigr ) + d\bigl (\varphi (t^*_{n_j};t^*_{n_j}-T,y_{n_j}),u\bigr ) \\&\quad = d\bigl (\varphi (t^*_{n_j};t^*_{n_j}-T,\bar{k}), \varphi (t^*_{n_j};t^*_{n_j}-T,y_{n_j})\bigr )\\&\qquad +d\bigl (\varphi (t^*_{n_j};t^*_{n_j}-T,\varphi (t^*_{n_j}-T;\tau _{n_j},a_{n_j})), u\bigr ) \\&\quad = d\bigl (\varphi (t^*_{n_j};t^*_{n_j}-T,\bar{k}), \varphi (t^*_{n_j};t^*_{n_j}-T,y_{n_j})\bigr ) + d\bigl (\varphi (t^*_{n_j};\tau _{n_j},a_{n_j}),u\bigr ) \\&\quad < \tfrac{\varepsilon }{2}+\tfrac{\varepsilon }{2}=\varepsilon \quad \text {for all }n\ge N_1(\varepsilon ),n_j\ge N_2(\varepsilon ,T). \end{aligned}$$

Setting $u_\varepsilon ^*{:}{=}\bar{k}\in \omega _{\mathcal {A}}^+$, we then obtain that $\omega _{\mathcal {A}}^+$ is asymptotically negatively invariant. $\square$

### Asymptotically Autonomous Difference Equations

In general it is difficult to obtain the forward limit set $\omega _{\mathcal {A}}^+$ given as limit of the fibres $\varOmega _{\mathcal {A}}(\tau )$ explicitly. This situation simplifies, if $(\varDelta )$ behaves asymptotically as an autonomous difference equation 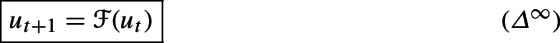 with right-hand side  in a sense to be specified below. Here, it is common to denote the iterates of  by , $s\in {\mathbb {N}}_0$. A maximal, invariant and nonempty compact set $A^*\subseteq U$ attracting all bounded subsets of *U* is called *global attractor* of $(\varDelta ^\infty )$ (cf. [8, p. 17]).

For a class of nonautonomous equations $(\varDelta )$ introduced next, the sets $\varOmega _{\mathcal {A}}(\tau )$, $\tau \in {\mathbb {I}}$, turn out to be constant and determined by the global attractor $A^*$ of $(\varDelta ^\infty )$.

#### Theorem 4.11

(Asymptotically autonomous difference equations) Suppose that $(\varDelta ^\infty )$ has a bounded absorbing set $A\subseteq U$ and a global attractor $A^*\subseteq A$. If ${\mathcal {A}}{:}{=}{\mathbb {I}}\times A$ is a forward absorbing set of $(\varDelta )$ and the condition4.9holds, then $\varOmega _{{\mathcal {A}}}(\tau )=A^*$ for all $\tau \in {\mathbb {I}}$ and in particular $\omega _{{\mathcal {A}}}^-=\omega _{{\mathcal {A}}}^+=A^*$.

#### Remark 4.12

Asymptotically autonomous difference equations were also studied in [5] in order to show that the fibres ${\mathcal {A}}^*(\tau )$ of a pullback attractor ${\mathcal {A}}^*$ to $(\varDelta )$ converge to the global attractor $A^*$ of the limit equation $(\varDelta ^\infty )$ as $\tau \rightarrow \infty$. In these results, however, asymptotic autonomy is based on e.g. the limit relation(see [5, Thm. 1]) with sequences $(a_n)_{n\in {\mathbb {N}}}$ converging to some $a_0$. This condition is clearly different from (4.9).

#### Proof

Given any $\tau \in {\mathbb {I}}$, we have to show two inclusions:

$(\subseteq )$ Let $v\in \varOmega _{\mathcal {A}}(\tau )$. Due to Remark 4.3 there exist sequences $a_n\in A$ and $s_n\rightarrow \infty$ as $n\rightarrow \infty$ with

$$\begin{aligned} \lim _{n\rightarrow \infty }\varphi (\tau +s_n;\tau ,a_n)=v \end{aligned}$$

and it follows from (4.9) thatsince the global attractor $A^*$ of $(\varDelta ^\infty )$ attracts the absorbing set *A*. This implies that $v\in A^*$, and since *v* was arbitrary, the inclusion $\varOmega _{\mathcal {A}}(\tau )\subseteq A^*$ holds for $\tau \in {\mathbb {I}}$.

$(\supseteq )$ Conversely, since $A^*$ is compact, there exists an $a^*\in A^*$ with

$$\begin{aligned} {\text {dist}}\bigl (A^*,\varOmega _{{\mathcal {A}}}(\tau )\bigr )&= {\text {dist}}\bigl (a^*,\varOmega _{{\mathcal {A}}}(\tau )\bigr )\\&\le {\text {dist}}\bigl (a^*,\varphi (\tau +s;\tau ,A^*)\bigr )+{\text {dist}}\bigl (\varphi (\tau +s;\tau ,A^*),\varOmega _{\mathcal {A}}(\tau )\bigr ) \end{aligned}$$

for all $s\in {\mathbb {I}}$ and we separately estimate the two terms on the right-hand side of this inequality. First, due to the invariance of $A^*$ there exists $a_s^*\in A^*$ with  and thereforeSecond, from $A^*\subseteq A={\mathcal {A}}(\tau )$ one has

$$\begin{aligned} {\text {dist}}\bigl (\varphi (\tau +s;\tau ,A^*),\varOmega _{\mathcal {A}}(\tau )\bigr ) \le {\text {dist}}\bigl (\varphi (\tau +s;\tau ,{\mathcal {A}}(\tau )),\varOmega _{\mathcal {A}}(\tau )\bigr ) \xrightarrow [s\rightarrow \infty ]{(4.2)}0, \end{aligned}$$

which guarantees the remaining inclusion $A^*\subseteq \varOmega _{\mathcal {A}}(\tau )$.

Hence, all $\varOmega _{\mathcal {A}}(\tau )$ are constant, thus $\omega _{\mathcal {A}}^-=A^*$ and $\omega _{\mathcal {A}}^+=\overline{A^*}=A^*$. $\square$

The following simple example illustrates the condition (4.9):

#### Example 4.13

(Beverton–Holt equation) If $\alpha >1$, then it is well known that all solutions to the autonomous Beverton–Holt equation $v_{t+1}=\tfrac{\alpha v_t}{1+v_t}$ starting with a positive initial value converge to $\alpha -1$ (see [18, pp. 13ff]). We establish that an asymptotically autonomous, but nonautonomous Beverton–Holt equation

4.10 $$\begin{aligned} v_{t+1}&=\frac{\tilde{a}_tv_t}{1+v_t},&\tilde{a}_t&{:}{=}\frac{f_{t+1}}{f_t}\alpha \end{aligned}$$

shares this behaviour, whenever the sequence $(f_t)_{t\in {\mathbb {I}}}$ in $(0,\infty )$ satisfies

4.11 $$\begin{aligned} \lim _{t\rightarrow \infty }\sum _{s=0}^{t-1}\frac{f_{\tau +s}}{f_{\tau +t}}\alpha ^{s-t}=\frac{1}{\alpha -1} \quad \text {for all }\tau \in {\mathbb {I}} \end{aligned}$$

and grows at most polynomially. For instance, the relation (4.11) holds for the sequences $f_t=(t+c)^n$ with $c\in {\mathbb {R}}$, $n\in \left\{ 1,2,3,4\right\}$. Indeed, the explicit representation

$$\begin{aligned} \varphi (\tau +t;\tau ,a) = \frac{(\alpha -1)a}{\tfrac{\alpha -1}{\alpha ^t}\tfrac{f_\tau }{f_{\tau +t}}+(\alpha -1)a\sum _{s=0}^{t-1}\tfrac{f_{\tau +s}}{f_{\tau +t}}\alpha ^{s-t}} \end{aligned}$$

of the general solution to (4.10) yields that $\lim _{t\rightarrow \infty }\varphi (t+\tau ;\tau ,a)=\alpha -1$ holds uniformly in $a\in [\bar{a},\infty )$ for any $\bar{a}>0$. Consequently, one has

$$\begin{aligned} \sup _{a\ge \bar{a}}\left| \varphi (\tau +t;\tau ,a)-F^t(a)\right| \le&\sup _{a\ge \bar{a}}\left| \varphi (\tau +t;\tau ,a)-(\alpha -1)\right| + \sup _{a\ge \bar{a}}\left| \alpha -1-F^t(a)\right| \\&\xrightarrow [t\rightarrow \infty ]{}0 \end{aligned}$$

and therefore (4.9) is valid with arbitrary subsets $A\subseteq [\bar{a},\infty )$.

We continue with two sufficient criteria for the condition (4.9) to hold. Thereto we assume in the remaining subsection that $(X,\left\| \cdot \right\| )$ is a Banach space.

#### Theorem 4.14

(Asymptotically autonomous linear difference equations) Suppose that , $b_t,b\in X$, $t\in {\mathbb {I}}$, satisfy4.12If , then $(\varDelta ^\infty )$ with right-hand side  and $(\varDelta )$ with right-hand side  fulfill the limit relation (4.9) on every bounded subset $A\subseteq X$.

#### Proof

We note that (4.12) implies that $(\varDelta )$ is uniformly exponentially stable and that the sequences , $(b_t)_{t\in {\mathbb {I}}}$ are bounded. Thus, [2, Cor. 5 with $H=\ell ^\infty ({\mathbb {I}},X)$] implies that $\varphi (\cdot ;\tau ,0)$ is bounded and the representation $\varphi (t;\tau ,u_\tau )=\varPhi (t,\tau )u_\tau +\varphi (t;\tau ,0)$ for all $t\ge \tau$, $u_\tau \in X$ shows that $\varphi (\cdot ;\tau ,u_\tau )$ is bounded uniformly in $u_\tau$ from bounded subsets of *X*. Now it is easy to see that the difference  solves the initial value problemwhose inhomogeneity  satisfies $\lim _{t\rightarrow \infty }\tilde{b}_t=0$ uniformly in *a* from bounded subsets of *X*. Now using [2, Cor. 5 with $H=c_0({\mathbb {I}},X)$] guarantees that the sequence  converges to 0 as $t\rightarrow \infty$ uniformly in *a* from bounded subsets of *X*, that is, in particular (4.9) holds. $\square$

#### Theorem 4.15

(Asymptotically autonomous semilinear difference equations) Let  be of semilinear form (2.5) such that

4.13 $$\begin{aligned} \left\| \varPhi (t,s)\right\| \le K\alpha ^{t-s}\quad \text {for all }s\le t \end{aligned}$$

and4.14with $K\ge 1$, $\alpha \in (0,1)$ and $L\in (0,\tfrac{1-\alpha }{K})$. If  and  satisfy

(i) there exists a $K_1\ge 0$ such that  for all $t\in {\mathbb {I}}$,
(ii) for every $r>0$ there exists a $K_2(r)\ge 1$ such that

then $(\varDelta ^\infty )$ with right-hand side  and $(\varDelta )$ fulfill the limit relation (4.9) even exponentially on every bounded subset $A\subseteq X$.

The assumption (4.13) holds in case  with  (see [22, Thm. 5]).

#### Proof

We proceed in two steps:

(I) Claim: *All solutions to the autonomous equation* $(\varDelta ^\infty )$ *are bounded, i.e.,*4.15This is a consequence of [20, p. 155, Thm. 3.5.8(a)].

(II) Let $a\in U$ and we abbreviate $u_t{:}{=}\varphi (t;\tau ,a)$, . Due to step (I) the sequence $(v_t)_{t\ge \tau }$ is bounded and we choose $r>0$ so large that $\left\| v_t\right\| <r$ for all $t\in {\mathbb {I}}$. It is easy to see that the difference $w_t{:}{=}u_t-v_t$ satisfies the equation4.16and fulfills the initial condition $u_\tau -v_\tau =a-a=0$. Using the variation of constants formula [20, p. 100, Thm. 3.1.16] resultsconsequentlyand the Grönwall inequality [20, p. 348, Prop. A.2.1(a)] yieldsIf we replace *t* by $\tau +t$, then it resultsand therefore even exponential convergence holds in (4.9). $\square$

### Forward Attractors

In the previous Sect. 3.2 we constructed pullback attractors of nonautonomous difference equations $(\varDelta )$ by means of Proposition 3.1 applied to a pullback absorbing, positively invariant nonautonomous set. Now it is our goal is to obtain a corresponding concept in forward time.

Mimicking the approach for pullback attractors, we define a *forward attractor* ${\mathcal {A}}^+\subset {\mathcal {U}}$ of $(\varDelta )$ as a nonempty, compact and invariant nonautonomous set forward attracting every bounded subset ${\mathcal {B}}\subseteq {\mathcal {U}}$, i.e.,

4.17 $$\begin{aligned} \lim _{s\rightarrow \infty }{\text {dist}}\bigl (\varphi (\tau +s;\tau ,{\mathcal {B}}(\tau )),{\mathcal {A}}^+(\tau +s)\bigr )=0 \quad \text {for all }\tau \in {\mathbb {I}}. \end{aligned}$$

As demonstrated in e.g. [16, Sect. 4], forward attractors need not to be unique. They are Lyapunov asymptotically stable, that is, Lyapunov stable and attractive in the sense of (4.17) (see [12, Prop. 3.1]). While it is often claimed in the literature that there is no counterpart to the characterisation (3.5) of pullback attractors ${\mathcal {A}}^*$ for forward attractors ${\mathcal {A}}^+$ of nonautonomous equations, a suitable construction will be given now.

A nonautonomous difference equation $(\varDelta )$ is denoted as *forward dissipative*, if there exists a bounded set ${\mathcal {A}}\subseteq {\mathcal {U}}$ such that for every $\tau \in {\mathbb {I}}$ and bounded ${\mathcal {B}}\subseteq {\mathcal {U}}$ there is an *absorption time* $S=S(\tau ,{\mathcal {B}})\in {\mathbb {N}}$ such that

4.18 $$\begin{aligned} \varphi (\tau +s;\tau ,{\mathcal {B}}(\tau ))\subseteq {\mathcal {A}}(\tau +s)\quad \text {for all }s\ge S; \end{aligned}$$

one says that ${\mathcal {A}}$ is as a *forward absorbing* set.

#### Proposition 4.16

(Forward absorbing set) On a Banach space *X*, let  be of the semilinear form (2.5) satisfying (2.7), (2.8) and let $\rho >0$. If the limit relations

$$\begin{aligned} \lim _{s\rightarrow \infty }\prod _{r=\tau }^{\tau +s-1}(\alpha _r+Ka_r)&=0,&R_\tau&{:}{=}K\lim _{t\rightarrow \infty }\sum _{s=\tau }^{t-1}b_s\prod _{r=s+1}^{t-1}(\alpha _r+Ka_r)<\infty \end{aligned}$$

hold for all $\tau \in {\mathbb {I}}$ and $\sup _{\tau \in {\mathbb {I}}}R_\tau <\infty$, then the difference equation $(\varDelta )$ is forward dissipative with absorbing set ${\mathcal {A}}{:}{=}\left\{ (t,u)\in {\mathcal {U}}:\,\left\| u\right\| \le \rho +\sup _{\tau \in {\mathbb {I}}}R_\tau \right\}$.

For constant positive sequences $\alpha _t\equiv \alpha$, $a_t\equiv a$, $b_t\equiv b$ in (2.8) satisfying $\alpha +Ka<1$, both the pullback absorbing set from Proposition 3.2 and the forward absorbing set from Proposition 4.16 have constant fibres and simplify to ${\mathcal {A}}={\mathbb {I}}\times B_{\rho +\tfrac{Kb}{1-\alpha -aK}}(0)$.

#### Proof

The claim follows readily from relation (2.9) in Lemma 2.1. $\square$

Using [12, Prop. 3.2 with compact replaced by open and bounded] one shows

#### Proposition 4.17

Every bounded forward attractor has a nonempty, positively invariant, closed and bounded forward absorbing set.

First, this Proposition 4.17 allows us to choose a closed and bounded, positively invariant set ${\mathcal {A}}\subseteq {\mathcal {U}}$. We then deduce a nonempty, invariant and compact nonautonomous set ${\mathcal {A}}^\star \subseteq {\mathcal {A}}$ from Proposition 3.1.

Second, the construction of forward attractors requires ${\mathbb {I}}={\mathbb {Z}}$. Different from the pullback situation (with ${\mathcal {A}}$ being pullback absorbing), having an forward absorbing set ${\mathcal {A}}$ does not ensure the forward convergence within ${\mathcal {A}}$, i.e.,

$$\begin{aligned} \lim _{s\rightarrow \infty }{\text {dist}}\bigl (\varphi (\tau +s;\tau ,{\mathcal {A}}(\tau )),{\mathcal {A}}^\star (\tau )\bigr )=0\quad \text {for all }\tau \in {\mathbb {Z}}\end{aligned}$$

and in particular not forward convergence of a general bounded nonautonomous set ${\mathcal {B}}\subseteq {\mathcal {U}}$ to ${\mathcal {A}}^*$. This is because $(\varDelta )$ may have forward limit points starting in ${\mathcal {A}}$ which are not forward limit points from within ${\mathcal {A}}^\star$. Corresponding examples illustrating this are given in [14].

Now on the one hand, the set of forward $\omega$-limit points for the dynamics starting in ${\mathcal {A}}^\star$ is given by

$$\begin{aligned} \omega _{{\mathcal {A}}}^\star {:}{=} \bigcap _{\tau \in {\mathbb {Z}}} \overline{\bigcup _{0\le s}\varphi (\tau +s;\tau ,{\mathcal {A}}^\star (\tau ))} = \bigcap _{\tau \in {\mathbb {Z}}}\overline{\bigcup _{0\le s}{\mathcal {A}}^\star (\tau +s)} \subseteq U \end{aligned}$$

and is nonempty and compact as intersection of nested compact sets. It consists of all points $u\in U$ such that there is a sequence $\bigl ((s_n,a_n)\bigr )_{n\in {\mathbb {N}}}$ with $\lim _{n\rightarrow \infty }s_n=\infty$ and $a_n\in {\mathcal {A}}(\tau +s_n)$ with $\tau \in {\mathbb {Z}}$ satisfying

$$\begin{aligned} \lim _{n\rightarrow \infty }\varphi (\tau +s_n;\tau ,a_n)=u. \end{aligned}$$

On the other hand, the set of forward limit points $\omega _{{\mathcal {A}}}^+$ from within ${\mathcal {A}}$ was constructed in Theorem 4.6. With ${\mathcal {A}}$ being positively invariant, the chain of inclusions $\omega _{{\mathcal {A}}}^-\subseteq \omega _{{\mathcal {A}}}^+\subseteq K$ holds, while $\omega _{{\mathcal {A}}}^\star$ is not necessarily contained in $\omega _{\mathcal {A}}^-$ (see Example 4.20 for an illustration), as well as

$$\begin{aligned} \lim _{t\rightarrow \infty }{\text {dist}}\bigl ({\mathcal {A}}^\star (t),\omega _{{\mathcal {A}}}^-\bigr )=0. \end{aligned}$$

#### Theorem 4.18

Suppose that $(\varDelta )$ has a positively invariant, closed and bounded set ${\mathcal {A}}\ne \emptyset$. If the assumptions in Proposition 3.1 and Theorems 4.9–4.10 hold, then the following statements are equivalent:

- ${\mathcal {A}}^\star$ is *forward attracting* ${\mathcal {A}}$, that is,
  4.19 $$\begin{aligned} \lim _{s\rightarrow \infty }{\text {dist}}\bigl (\varphi (\tau +s;\tau ,{\mathcal {A}}(\tau )),{\mathcal {A}}^\star (\tau +s)\bigr )=0\quad \text {for all }\tau \in {\mathbb {Z}}, \end{aligned}$$
- $$\omega _{{\mathcal {A}}}^+=\omega _{{\mathcal {A}}}^\star $$.

#### Proof

($\Rightarrow$) Suppose that ${\mathcal {A}}^\star$ is forward attracting from within ${\mathcal {A}}$ and that $\omega _{{\mathcal {A}}}^+\ne \omega _{{\mathcal {A}}}^\star$. Since $\omega _{{\mathcal {A}}}^\star \subseteq \omega _{{\mathcal {A}}}^+$, there exists a point $\tilde{v}\in \omega _{{\mathcal {A}}}^+\setminus \omega _{{\mathcal {A}}}^\star$, i.e., there are $\tilde{\tau }\in {\mathbb {Z}}$ and $\tilde{\varepsilon }=\tilde{\varepsilon }(\tilde{\tau })>0$ such that $\tilde{v}\in \varOmega _{\mathcal {A}}(\tilde{\tau })$ and

4.20 $$\begin{aligned} {\text {dist}}\bigl (\tilde{v},\omega _{{\mathcal {A}}}^\star \bigr )> 2\tilde{\varepsilon }. \end{aligned}$$

Since $\tilde{v}\in \varOmega _{\mathcal {A}}(\tilde{\tau })$, there exists a sequence $\bigl ((\tilde{s}_n,\tilde{b}_n)\bigr )_{n\in {\mathbb {N}}}$ with $\lim _{n\rightarrow \infty }\tilde{s}_n=\infty$ and points $\tilde{b}_n\in {\mathcal {A}}(\tilde{\tau })$ satisfying ${\text {dist}}\bigl (\tilde{v},\varphi (\tilde{\tau }+\tilde{s}_n;\tilde{\tau },\tilde{b}_n)\bigr )<\tilde{\varepsilon }$. Moreover, by the forward attraction of ${\mathcal {A}}^\star$, there exists an $s'>0$ such that

$$\begin{aligned} {\text {dist}}\bigl (\varphi (\tilde{\tau }+s';\tilde{\tau },{\mathcal {A}}(\tilde{\tau })),{\mathcal {A}}^\star (\tilde{\tau }+s')\bigr )<\tilde{\varepsilon }. \end{aligned}$$

Combining all of them, we obtain

$$\begin{aligned} {\text {dist}}\bigl (\tilde{v},{\mathcal {A}}^\star (\tilde{\tau }+\tilde{s}_n)\bigr )\le & {} {\text {dist}}\bigl (\tilde{v},\varphi (\tilde{\tau }+\tilde{s}_n;\tilde{\tau },\tilde{b}_n)\bigr ) + {\text {dist}}\bigl (\varphi (\tilde{\tau }+\tilde{s}_n;\tilde{\tau },\tilde{b}_n),{\mathcal {A}}^\star (\tau +\tilde{s}_n)\bigr )\\\le & {} {\text {dist}}\bigl (\tilde{v},\varphi (\tilde{\tau }+\tilde{s}_n;\tilde{\tau },\tilde{b}_n)\bigr ) + {\text {dist}}\bigl (\varphi (\tilde{\tau }+\tilde{s}_n;\tilde{\tau },{\mathcal {A}}(\tilde{\tau })),{\mathcal {A}}^\star (\tau +\tilde{s}_n)\bigr )\\< & {} \tilde{\varepsilon }+\tilde{\varepsilon }=2\tilde{\varepsilon }. \end{aligned}$$

Since $\bigcap _{n\in {\mathbb {N}}}\overline{\bigcup _{m\ge n}{\mathcal {A}}^\star (\tilde{\tau }+\tilde{s}_n)}\subseteq \omega _{{\mathcal {A}}}^\star$ by definition, it then follows

$$\begin{aligned} {\text {dist}}\bigl (\tilde{v},\omega _{{\mathcal {A}}}^\star \bigr ) \le {\text {dist}}\left( \tilde{v},\bigcap _{n\in {\mathbb {N}}}\overline{\bigcup _{m\ge n} {\mathcal {A}}^\star (\tilde{\tau }+\tilde{s}_n)}\right) \le {\text {dist}}\bigl (\tilde{v},{\mathcal {A}}^\star (\tilde{\tau }+\tilde{s}_n)\bigr ) < 2\tilde{\varepsilon }, \end{aligned}$$

a contradiction to (4.20). Hence, $\omega _{{\mathcal {A}}}^+=\omega _{{\mathcal {A}}}^\star$ holds.

($\Leftarrow$) Suppose that $\omega _{{\mathcal {A}}}^+=\omega _{{\mathcal {A}}}^\star$, i.e., ${\text {dist}}\bigl (\omega _{{\mathcal {A}}}^+,\omega _{{\mathcal {A}}}^\star \bigr )<\varepsilon$ for all $\varepsilon >0$, and that ${\mathcal {A}}^\star$ is not forward attracting from within ${\mathcal {A}}$, i.e., there exist a real $\tilde{\varepsilon }>0$ and a sequence $(\tilde{s}_n)_{n\in {\mathbb {N}}}$ in ${\mathbb {N}}_0$ with $\lim _{n\rightarrow \infty }\tilde{s}_n=\infty$ and

$$\begin{aligned} {\text {dist}}\bigl (\varphi (\tau +\tilde{s}_n;\tau ,{\mathcal {A}}^\star (\tau )),{\mathcal {A}}(\tau +\tilde{s}_n)\bigr )\ge 2\tilde{\varepsilon } \quad \text {for all }n\in {\mathbb {N}}. \end{aligned}$$

Although there is no condition ensuring the set $\varphi (\tau +s_n;\tau ,{\mathcal {A}}(\tau ))$ is compact, which means the supremum in the Hausdorff semidistance may not be attained, there still exists a point $\tilde{y}_n{:}{=}\varphi (\tau +s_n;\tau ,\tilde{b}_n) \in \varphi (\tau +s_n;\tau ,{\mathcal {A}}(\tau ))$ for all $n\in {\mathbb {N}}$ and $\tilde{b}_n\in {\mathcal {A}}(\tau )$ such that

$$\begin{aligned} {\text {dist}}\bigl (\varphi (\tau +\tilde{s}_n;\tau ,{\mathcal {A}}(\tau )),{\mathcal {A}}^\star (\tau +\tilde{s}_n)\bigr )-\tilde{\varepsilon }&\le {\text {dist}}\bigl (\tilde{y}_n,{\mathcal {A}}^\star (\tau +\tilde{s}_n)\bigr )\\&\le {\text {dist}}\bigl (\varphi (\tau +\tilde{s}_n;\tau ,{\mathcal {A}}(\tau )\bigr ),{\mathcal {A}}^\star (\tau +\tilde{s}_n)). \end{aligned}$$

The above inequalities in fact give ${\text {dist}}\bigl (\tilde{y}_n,{\mathcal {A}}^\star (\tau +\tilde{s}_n)\bigr )\ge \tilde{\varepsilon }$ for all $n\in {\mathbb {N}}$. Moreover, take a point $\tilde{a}_n\in {\mathcal {A}}^\star (\tau +\tilde{s}_n)$, then

$$\begin{aligned} d(\tilde{y}_n,\tilde{a}_n)\ge {\text {dist}}\bigl (\tilde{y}_n,{\mathcal {A}}(\tau +\tilde{s}_n)\bigr )\ge \tilde{\varepsilon } \quad \text {for all }n\in {\mathbb {N}}. \end{aligned}$$

On the other hand, by assumptions and definitions, $(\varDelta )$ is ${\mathcal {A}}$-asymptotically compact and both $\tilde{y}_n$ and $\tilde{a}_n$ are in $\varphi (\tau +s_n;\tau ,{\mathcal {A}}(\tau ))$ for all $\tau \in {\mathbb {Z}}$, so

$$\begin{aligned} {\text {dist}}\bigl (\tilde{y}_n,K\bigr )&\le {\text {dist}}\bigl (\varphi (\tau +\tilde{s}_n;\tau ,{\mathcal {A}}(\tau )),K\bigr ) \xrightarrow [n\rightarrow \infty ]{}0,\\ {\text {dist}}\bigl (\tilde{a}_n,K\bigr )&\le {\text {dist}}\bigl (\varphi (\tau +\tilde{s}_n;\tau ,{\mathcal {A}}(\tau )),K\bigr ) \xrightarrow [n\rightarrow \infty ]{}0, \end{aligned}$$

implying that both $\tilde{y}_n$ and $\tilde{a}_n$ are in *K* as well. Additionally, since *K* is compact, there are convergent subsequences $(\tilde{y}_{n_j})_{j\in {\mathbb {N}}}$ with limit $\tilde{y}\in K$ and $(\tilde{a}_{n_j})_{j\in {\mathbb {N}}}$ with limit $\tilde{a}\in K$. This implies $\tilde{y}\in \varOmega _{\mathcal {A}}(\tau )\subseteq \omega _{{\mathcal {A}}}^+$ and $\tilde{a}\in \omega _{{\mathcal {A}}}^\star$ by definitions. Combining this with $d(\tilde{y}_n,\tilde{a}_n)\ge \tilde{\varepsilon }$ for all $n\in {\mathbb {N}}$, we arrive at the contradiction

$$\begin{aligned} {\text {dist}}\bigl (\omega _{{\mathcal {A}}}^+,\omega _{{\mathcal {A}}}^\star \bigr )\ge {\text {dist}}\bigl (\tilde{y}_n,\omega _{{\mathcal {A}}}^\star \bigr )\ge d(\tilde{y},\tilde{a})\ge \tilde{\varepsilon } \quad \text {for all }n\in {\mathbb {N}}, \end{aligned}$$

to the assumption. Thus, ${\mathcal {A}}^\star$ is forward attracting from within ${\mathcal {A}}$. $\square$

#### Corollary 4.19

Suppose in addition that ${\mathcal {A}}\subseteq {\mathcal {U}}$ is forward absorbing. If $\omega _{{\mathcal {A}}}^+=\omega _{{\mathcal {A}}}^\star$ holds, then ${\mathcal {A}}^\star$ is a forward attractor of $(\varDelta )$.

#### Proof

Due to Proposition 3.1 the set ${\mathcal {A}}^\star$ is already nonempty, compact, invariant and thus it suffices to show that ${\mathcal {A}}^\star$ is forward attracting. Thereto, suppose that ${\mathcal {B}}\subseteq {\mathcal {U}}$ is bounded and choose $\tau \in {\mathbb {Z}}$ arbitrarily. With the forward absorption time $S\in {\mathbb {N}}$ we obtain from Theorem 4.18 that

$$\begin{aligned} 0\le & {} {\text {dist}}\bigl (\varphi (\tau +s;\tau ,{\mathcal {B}}(\tau )),{\mathcal {A}}^\star (\tau +s)\bigr )\\&{\mathop {=}\limits ^{(2.3)}}&{\text {dist}}\bigl (\varphi \bigl (\tau +s;\tau +S,\varphi (\tau +S,\tau ,{\mathcal {B}}(\tau ))\bigr ),{\mathcal {A}}^\star (\tau +s)\bigr )\\&{\mathop {\le }\limits ^{(4.18)}}&{\text {dist}}\bigl (\varphi (\tau +s;\tau +S,{\mathcal {A}}(\tau +S)),{\mathcal {A}}^\star (\tau +s)\bigr ) \xrightarrow [s\rightarrow \infty ]{(4.19)}0 \end{aligned}$$

and this yields the assertion. $\square$

We close this section with a simple, yet illustrative example:

#### Example 4.20

(Beverton–Holt equation) Given reals $0<\alpha _-,\alpha _+$ we consider the asymptotically autonomous Beverton–Holt equation

4.21 $$\begin{aligned} v_{t+1}&=\frac{\tilde{a}_tv_t}{1+v_t},&\tilde{a}_t&{:}{=} {\left\{ \begin{array}{ll} \alpha _-,&{}t<0,\\ \alpha _+,&{}0\le t \end{array}\right. } \end{aligned}$$

in $U={\mathbb {R}}_+$ having the general solution

$$\begin{aligned} \varphi (t;\tau ,v_\tau ) = \frac{v_\tau \prod _{r=\tau }^{t-1}\tilde{a}_r}{1+v_\tau \sum _{s=\tau }^{t-1}\prod _{r=\tau }^{s-1}\tilde{a}_r} \quad \text {for all }\tau \le t,\,0\le v_\tau . \end{aligned}$$

It possesses the absorbing set ${\mathcal {A}}={\mathbb {Z}}\times [0,\max \left\{ \alpha _-,\alpha _+\right\} +1]$ and the forward $\omega$-limit set $\omega _{\mathcal {A}}^+=[0,\max \left\{ 0,\alpha _+-1\right\} ]$. Depending on the constellation of the parameters $\alpha _-,\alpha _+$ one obtains the following capturing the forward dynamics:


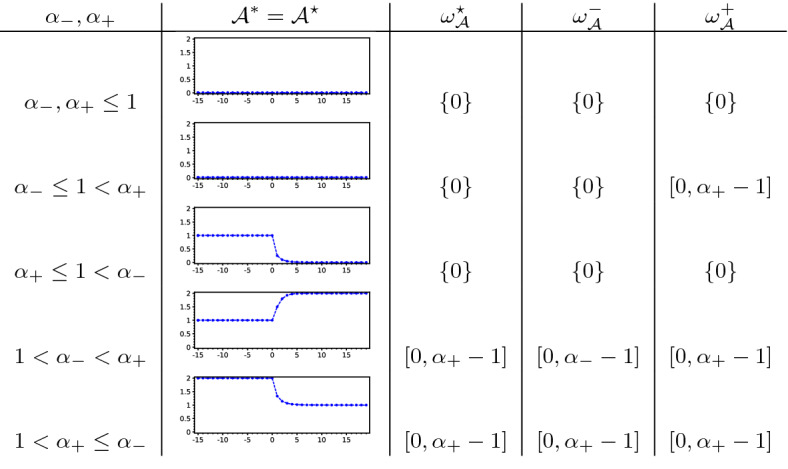


For $\alpha _+\le 1$ all fibres $\varOmega _{\mathcal {A}}(\tau )=\left\{ 0\right\}$ are constant. For $\alpha _+>1$ two cases arise:

- $1\le \alpha _-<\alpha _+$: Solutions starting in $\alpha _++1$ at time $\tau <0$ first decay until time $t=0$ and then increase again, which yields
  $$\begin{aligned} \varOmega _{{\mathcal {A}}}(\tau ) = {\left\{ \begin{array}{ll} {[}0,\varphi (0;\tau ,\alpha _++1)],&{}\tau <0,\\ {[}0,\alpha _+-1],&{}0\le \tau . \end{array}\right. } \end{aligned}$$
- $1\le \alpha _+\le \alpha _-$: Solutions starting in $\alpha _++1$ decay to $\alpha _+-1$ and thus the fibres are constant $\varOmega _{\mathcal {A}}(\tau )\equiv [0,\alpha _++1]$ on ${\mathbb {Z}}$.

Except for $\alpha _-\le 1<\alpha _+$, where the pullback and forward dynamics of (4.21) differ, Corollary 4.19 applies and yields that the pullback attractor ${\mathcal {A}}^*={\mathcal {A}}^\star$ is the forward attractor ${\mathcal {A}}^+$.

As a conclusion, in case ${\mathcal {A}}$ is a positively invariant, forward absorbing nonautonomous set this section provided two concepts to capture the forward dynamics of $(\varDelta )$, namely the limit set $\omega _{{\mathcal {A}}}^+$ from Theorem 4.6 and the forward attractor ${\mathcal {A}}^+={\mathcal {A}}^\star$ constructed in Corollary 4.19. On the one hand, the limit set $\omega _{{\mathcal {A}}}^+\subseteq U$ is asymptotically positively invariant, forward attracts and is contained in all other sets with these properties. It depends only on information in forward time. On the other hand, the forward attractor ${\mathcal {A}}^+\subseteq {\mathcal {U}}$ shares these properties, but is actually invariant. Its construction is based on information on the entire axis ${\mathbb {Z}}$ and more restrictively, relies on the condition $\omega _{{\mathcal {A}}}^+=\omega _{{\mathcal {A}}}^\star$ from Theorem 4.18(b). The latter might be hard to verify in concrete examples, unless rather strict assumptions like asymptotic autonomy hold [5].

## Integrodifference Equations

The above abstract results will now be applied to nonautonomous IDEs. For this purpose let $(\varOmega ,{\mathfrak {A}},\mu )$ be a measure space satisfying $\mu (\varOmega )<\infty$. Suppose additionally that $\varOmega$ is equipped with a metric such that it becomes a compact metric space.

We consider the Banach space $X=C(\varOmega ,{\mathbb {R}}^d)$ of continuous ${\mathbb {R}}^d$-valued functions over $\varOmega$ equipped with the norm $\left\| u\right\| _0{:}{=}\max _{x\in \varOmega }\left| u(x)\right| .$ If $Z\subseteq {\mathbb {R}}^d$ is a nonempty, closed set, then $U{:}{=}\bigl \{u:\varOmega \rightarrow Z\mid u\in C(\varOmega ,{\mathbb {R}}^d)\bigr \}$ is a complete metric space. Furthermore, we have ${\mathcal {U}}={\mathbb {I}}\times U$, where ${\mathbb {I}}$ is an unbounded discrete interval.

Given functions $g_t:\varOmega \times Z\rightarrow {\mathbb {R}}^d$ and $k_t:\varOmega ^2\times Z\rightarrow {\mathbb {R}}^d$, the *Nemytskii operator*  is defined byand the *Urysohn integral operators*  byWith these operators, a nonautonomous difference equation $(\varDelta )$ of the additive form (2.10) is called an *integrodifference equation* and explicitly reads as 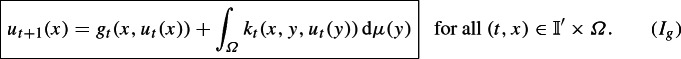 Such problems are well-motivated from applications:

- For an integrodifferential equation $D_1u(t,x)=\int _\varOmega f(t,x,y,u(t,y))\,{\mathrm {d}}y$ with, e.g., a continuous kernel function $f:{\mathbb {R}}\times \varOmega ^2\times {\mathbb {R}}^d\rightarrow {\mathbb {R}}^d$, the forward Euler discretisation with step-size $h>0$ gives the IDE
  $$\begin{aligned} u_{t+1}(x)=u_t(x)+h\int _\varOmega f(ht,x,y,u_t(y))\,{\mathrm {d}}y\quad \text {for all }(t,x)\in {\mathbb {I}}'\times \varOmega \end{aligned}$$
  matching ($I_g$) with a compact $\varOmega \subset {\mathbb {R}}^\kappa$ and the Lebesgue measure $\mu$.
- Population genetics or ecological models of the form
  $$\begin{aligned} u_{t+1}(x)=(1-\vartheta )g(x,u_t(x))+\vartheta \int _\varOmega f(x,y,u_t(y))\,{\mathrm {d}}y \quad \text {for all }(t,x)\in {\mathbb {I}}'\times \varOmega \end{aligned}$$
  are investigated in [25], where $\vartheta \in [0,1]$ is a parameter and e.g. continuous functions $g:\varOmega \times {\mathbb {R}}^d\rightarrow {\mathbb {R}}^d$, $f:\varOmega ^2\times {\mathbb {R}}^d\rightarrow {\mathbb {R}}^d$. These problems are of the from ($I_g$) with a compact $\varOmega \subset {\mathbb {R}}^\kappa$ and the Lebesgue measure $\mu$.
- Let the compact set $\varOmega \subset {\mathbb {R}}^\kappa$ be countable, $\eta \in \varOmega$ and $w_\eta \ge 0$ be reals. Then $\mu (\varOmega '){:}{=}\sum _{\eta \in \varOmega '}w_\eta$ defines a measure on the family of all countable subsets $\varOmega '\subset {\mathbb {R}}^\kappa$. The assumption $\sum _{\eta \in \varOmega }w_\eta <\infty$ ensures that $\mu (\varOmega )<\infty$. W.r.t. the resulting $\mu$-integral $\int _\varOmega u\,{\mathrm {d}}\mu =\sum _{\eta \in \varOmega }w_\eta u(\eta )$ the IDE ($I_g$) becomes
  $$\begin{aligned} u_{t+1}(x) = g_t(x,u_t(x)) + \sum _{\eta \in \varOmega }w_\eta k_t(x,\eta ,u_t(\eta )) \quad \text {for all }(t,x)\in {\mathbb {I}}'\times \varOmega . \end{aligned}$$
  Such difference equations occur as *Nyström methods* with *nodes* $\eta$ and *weights* $w_\eta$ as used in numerical discretizations and simulations [1] of IDEs ($I_g$).

**Hypothesis**: For every $t\in {\mathbb {I}}'$ we suppose:

$$(H_1)$$
The function $g_t:\varOmega \times Z\rightarrow {\mathbb {R}}^d$ is such that $g_t(\cdot ,z):\varOmega \rightarrow {\mathbb {R}}^d$ is continuous and there exist reals $\gamma _t,\ell _t\ge 0$ with
  $$\begin{aligned} \left| g_t(x,z)\right|&\le \gamma _t,&\left| g_t(x,z)-g_t(x,\bar{z})\right|&\le \ell _t\left| z-\bar{z}\right| \quad \text {for all }x\in \varOmega ,\,z,\bar{z}\in Z. \end{aligned}$$
$$(H_2)$$
The kernel function $k_t:\varOmega \times \varOmega \times Z\rightarrow {\mathbb {R}}^d$ is such that $k_t(x,\cdot ,z):\varOmega \rightarrow {\mathbb {R}}^d$ is measurable for all $x\in \varOmega$, $z\in Z$, and the following holds for almost all $y\in \varOmega$: $k_t(x,y,\cdot ):Z\rightarrow {\mathbb {R}}^d$ is continuous for all $x\in \varOmega$ and the limit
  $$\begin{aligned} \lim _{x\rightarrow x_0}\int _\varOmega \sup _{z\in Z\cap \bar{B}_r(0)}\left| k_t(x,y,z)-k_t(x_0,y,z)\right| \,{\mathrm {d}}\mu (y)=0 \quad \text {for all }r>0 \end{aligned}$$
  holds uniformly in $x_0\in \varOmega$.
$$(H_3)$$
There exists a function $\kappa _t:\varOmega ^2\rightarrow {\mathbb {R}}_+$, measurable in the second argument with $\sup _{x\in \varOmega }\int _\varOmega \kappa _t(x,y)\,{\mathrm {d}}\mu (y)<\infty$ and for almost all $y\in \varOmega$ one has
  $$\begin{aligned} \left| k_t(x,y,z)\right| \le \kappa _t(x,y)\quad \text {for all }x\in \varOmega ,\,z\in Z. \end{aligned}$$

Then the Nemytskii operator  satisfies5.1while the Urysohn operators  are globally bounded by5.2and completely continuous due to [19, p. 166, Prop. 3.2].

In the following we tacitly suppose  for all $t\in {\mathbb {I}}'$.

### Proposition 5.1

(Dissipativity for ($$I_g$$)) If $$(H_1$$–$$H_3)$$ with

5.3 $$\begin{aligned} \sup _{t\in {\mathbb {I}}'}\gamma _t&<\infty ,&\sup _{t\in {\mathbb {I}}'}\rho _t&<\infty \end{aligned}$$

hold, then the bounded and closed set

$$\begin{aligned} {\mathcal {A}}&{:}{=}\left\{ (t,u)\in {\mathcal {U}}:\,\left\| u\right\| _0\le \gamma _{t^*}+\rho _{t^*}\right\} ,&t^*&{:}{=} {\left\{ \begin{array}{ll} t-1,&{}t>\min {\mathbb {I}},\\ t,&{}t=\min {\mathbb {I}}\end{array}\right. } \end{aligned}$$

is positively invariant, forward absorbing (if ${\mathbb {I}}$ is unbounded above), pullback absorbing (if ${\mathbb {I}}$ is unbounded below) w.r.t. ($I_g$) with absorption time 1.

### Proof

Clearly the set ${\mathcal {A}}$ is closed and due to (5.3) also bounded. Let $t,\tau \in {\mathbb {I}}$ with $\tau <t$. Thus, $t^*=t-1$ and our assumptions readily imply thatand consequently $\varphi (t,\tau ,{\mathcal {U}}(\tau ))\subseteq {\mathcal {A}}(t)$ holds for all $\tau <t$. Thanks to ${\mathcal {A}},{\mathcal {B}}\subseteq {\mathcal {U}}$ for any bounded ${\mathcal {B}}$ this inclusion guarantees that ${\mathcal {A}}$ is positively invariant, but also forward and uniformly absorbing with absorption time $S=1$. $\square$

### Theorem 5.2

(Pullback attractor for ($$I_g$$)) Let $${\mathbb {I}}$$ be unbounded below. If $$(H_1$$–$$H_3)$$ are satisfied with (5.3) and there exists a $$T\in {\mathbb {I}}$$ such that $$\prod _{s=-\infty }^{T-1}\ell _s=0$$ hold, then the IDE ($$I_g$$) has a unique and bounded pullback attractor $${\mathcal {A}}^*\subseteq {\mathcal {A}}$$.

### Proof

We aim to apply Theorem 3.3 to ($I_g$). Thereto, Proposition 5.1 guarantees that ($I_g$) is uniformly pullback absorbing. Moreover, since the Lipschitz constant $\ell _t$ of the Nemytskii operator  is an upper bound for its Darbo constant and because  is completely continuous, the assertion follows. $\square$

Without further assumptions not much can be said about the detailed structure of the pullback attractor ${\mathcal {A}}^*$. Nevertheless, in case the functions $g_t,k_t$ satisfy monotonicity assumptions in the second resp. third argument, it is possible to construct “extremal” solutions in the attractor [21]. We illustrate this in

### Example 5.3

(spatial Beverton–Holt equation) Let $\vartheta \in [0,1]$ and $a_t:\varOmega \rightarrow (0,\infty )$, $t\in {\mathbb {I}}'$, be continuous functions describing the space- and time-dependent growth rates and a compact habitat $\varOmega$. The spatial Beverton–Holt equation

5.4 $$\begin{aligned} u_{t+1}(x) = (1-\vartheta ) \frac{a_t(x)u_t(x)}{1+u_t(x)} + \vartheta \int _\varOmega k(x,y)\frac{a_t(y)u_t(y)}{1+u_t(y)}\,{\mathrm {d}}\mu (y) \end{aligned}$$

for all $(t,x)\in {\mathbb {I}}'\times \varOmega$ fits into the framework of ($I_g$) with $Z={\mathbb {R}}_+$, $U=C(\varOmega ,{\mathbb {R}}_+)$,

$$\begin{aligned} g_t:\varOmega \times {\mathbb {R}}_+&\rightarrow {\mathbb {R}}_+,&g_t(x,z)&{:}{=}\frac{a_t(x)z}{1+z},\\ k_t:\varOmega \times \varOmega \times {\mathbb {R}}_+&\rightarrow {\mathbb {R}}_+,&k_t(x,y,z)&{:}{=}k(x,y)\frac{a_t(y)z}{1+z} \end{aligned}$$

and a continuous kernel function $$k:\varOmega \times \varOmega \rightarrow (0,\infty )$$. Then $$(H_1$$–$$H_3)$$ hold with

$$\begin{aligned} \gamma _t&=(1-\vartheta )\alpha _t,&\ell _t&=(1-\vartheta )\alpha _t,&\kappa _t(x,y)&=\vartheta \alpha _tk(x,y)\quad \text {for all }x,y\in \varOmega \end{aligned}$$

and $\alpha _t{:}{=}\max _{x\in \varOmega }a_t(x)$. If $\lim _{t\rightarrow -\infty }(1-\vartheta )^{T-t}\prod _{s=t}^{T-1}\alpha _s=0$ holds for some $T\in {\mathbb {I}}$, then Theorem 5.2 yields the existence of a pullback attractor ${\mathcal {A}}^*\subseteq {\mathcal {U}}$ for (5.4). Since the functions $g_t(x,\cdot ),k_t(x,y,\cdot ):{\mathbb {R}}_+\rightarrow {\mathbb {R}}_+$ are strictly increasing more can be said on the structure of ${\mathcal {A}}^*$. As in [21, Prop. 8], there exists an “extremal” entire solution $\phi ^+$ being pullback attracting from above such that

$$\begin{aligned} {\mathcal {A}}^*\subseteq \left\{ (t,u)\in {\mathcal {U}}:\,0\le u(x)\le \phi _t^+(x)\text { for all }x\in \varOmega \right\} . \end{aligned}$$

**Fig. 1:**
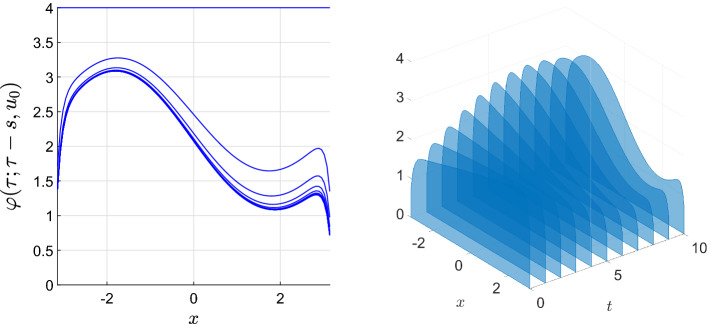
Pullback convergence to the fibre $\phi _\tau ^+:\varOmega \rightarrow {\mathbb {R}}_+$ ($\tau =10$, initial function $u_0(x)\equiv 4$, left) and sequence of sets containing the pullback attractor ${\mathcal {A}}^*$ ($0\le t\le 10$, right) for $\vartheta =\tfrac{1}{4}$

We illustrate both the pullback convergence to the solution $\phi ^+$, as well as the sets containing solutions in the pullback attractor ${\mathcal {A}}^*$ in Fig. 1, where $\varOmega =[-\pi ,\pi ]$ is equipped with the 1-dimensional Lebesgue measure, $a_t(x){:}{=}3-\sin \tfrac{tx}{10}$ (artificial) and the Laplace kernel $k(x,y){:}{=}\tfrac{a}{2}e^{-a\left| x-y\right| }$ for the dispersal rate $a=10$.

The remaining section addresses forward attraction. For simplicity we restrict to the class of Urysohn IDEs 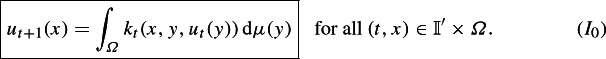


**Hypothesis**: For every $t\in {\mathbb {I}}'$ we suppose:

$$(H_4)$$
For all $r>0$ there exists a function $\lambda _t:\varOmega ^2\rightarrow {\mathbb {R}}_+$, measurable in the second argument with $\sup _{x\in \varOmega }\int _\varOmega \lambda _t(x,y)\,{\mathrm {d}}\mu (y)<\infty$ and for almost all $y\in \varOmega$ one has
  $$\begin{aligned} \left| k_t(x,y,z)-k_t(x,y,z)\right| \le \lambda _t(x,y)\left| z-\bar{z}\right| \quad \text {for all }x\in \varOmega ,\,z,\bar{z}\in Z\cap \bar{B}_r(0). \end{aligned}$$

### Proposition 5.4

(Dissipativity for ($$I_0$$)) If $$(H_2$$–$$H_3)$$ with $$R{:}{=}\sup _{t\in {\mathbb {I}}}\rho _t<\infty $$ hold, then the bounded and compact nonautonomous setis positively invariant, forward absorbing w.r.t. ($$I_0$$) with absorption time 2.

### Proof

Let $t\in {\mathbb {I}}'$ with $t-1\in {\mathbb {I}}'$ and thus $t^*=t-1$. Since the Urysohn operators  are completely continuous, the fibres  are compact. Thanks toit follows that ${\mathcal {A}}$ is bounded. Moreover,  holds for all $t\in {\mathbb {I}}'$ and ${\mathcal {A}}$ is positively invariant. Furthermore, from the inclusionwe deduce that ${\mathcal {A}}$ is absorbing. $\square$

### Theorem 5.5

(Forward limit set for ($$I_0$$)) Suppose that $$(H_2$$–$$H_3)$$ hold with additionally $$R{:}{=}\sup _{t\in {\mathbb {I}}}\rho _t<\infty $$. If  is relatively compact and $${\mathcal {A}}$$ is the forward absorbing set from Proposition 5.4, then the following are true:

- $\omega _{{\mathcal {A}}}^+$ is asymptotically positively invariant,
- $\omega _{{\mathcal {A}}}^+$ is asymptotically negatively invariant, provided $(H_4)$ is satisfied with
  5.5 $$\begin{aligned} \sup _{\tau \le t<\tau +T}\prod _{s=\tau }^{t-1} \int _\varOmega \lambda _s(x,y)\,{\mathrm {d}}\mu (y)<\infty \quad \text {for all }\tau \in {\mathbb {I}},\,T\in {\mathbb {N}}. \end{aligned}$$

The relative compactness of the union  holds for instance, if the kernel functions $k_t$ stem from a finite set or the images  form a nonincreasing/nondecreasing sequence of sets.

### Proof

By assumption the set  is compact and this implies that ($I_0$) is strongly ${\mathcal {A}}$-asymptotically compact.

(a) By construction of $K\subseteq U$ the assertion results from Theorem 4.9.

(b) Let $u,\bar{u}\in {\mathcal {A}}(s)\cup K$ and choose $r>0$ so large that ${\mathcal {A}}(s)\cup K\subseteq B_r(0)$. We concludefrom assumption $(H_4)$. After passing to the least upper bound over $x\in \varOmega$ it follows that $\sup _{x\in \varOmega }\int _\varOmega \lambda _s(x,y)\,{\mathrm {d}}\mu (y)$ is a Lipschitz constant for  on ${\mathcal {A}}(s)\cup K$. Hence, the assumption (5.5) implies (4.8) and therefore Theorem 4.10 yields the claim. $\square$

The above results do apply to the following

### Example 5.6

(spatial Ricker equation) Suppose that the compact $\varOmega \subseteq {\mathbb {R}}^\kappa$ is equipped with the $\kappa$-dimensional Lebesgue measure $\mu$ and that $\mu (\varOmega )>0$ holds. Let $(\alpha _t)_{t\in {\mathbb {I}}'}$ denote a bounded sequence of positive reals, $k:\varOmega \times \varOmega \rightarrow {\mathbb {R}}_+$ be continuous and $(b_t)_{t\in {\mathbb {I}}'}$ be a bounded sequence in $C(\varOmega ,{\mathbb {R}}_+)$, $t\in {\mathbb {I}}'$. The spatial Ricker equation

**Fig. 2:**
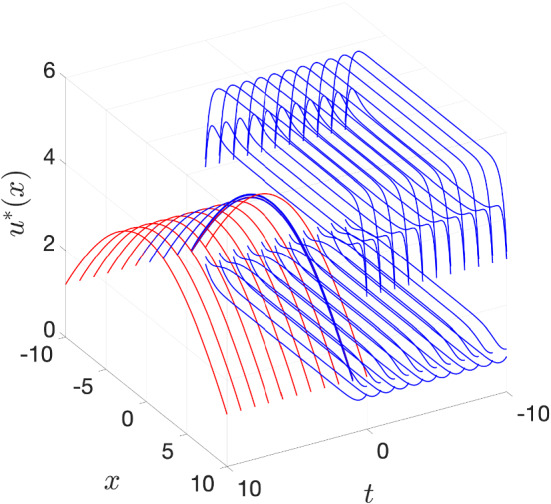
Functions contained in the fibres ${\mathcal {A}}^*(t)$ of the pullback attractor over the times $-10\le t\le 10$ (blue) and the forward limit set ${\mathbb {N}}_0\times \omega _{{\mathcal {A}}}^+={\mathbb {N}}_0\times \left\{ u^*\right\}$ (red) for the spatial Ricker equation (5.6) with Laplace kernel ($a=2$, $L=10$), $\alpha _+=0.12$, $\alpha _-=14$ and the constant inhomogeneity $b(x){:=}5\cos \tfrac{x}{8}$. More detailed, depicted are the 4-periodic orbits (blue) of the spatial Ricker equation, which is autonomous for $t<0$. In addition, the fibres ${\mathcal {A}}^*(t)$ also contain 0, a nontrivial fixed point and a 2-periodic orbit (Color figure online)

5.6 $$\begin{aligned} u_{t+1}(x) = \alpha _t \int _\varOmega k(x,y)u_t(y)e^{-u_t(y)}\,{\mathrm {d}}y+b_t(x) \quad \text {for all }(t,x)\in {\mathbb {I}}'\times \varOmega \end{aligned}$$

fits in the framework of ($I_0$) with $Z={\mathbb {R}}_+$ and the kernel function

$$\begin{aligned} k_t(x,y,z){:}{=}\alpha _tk(x,y)ze^{-z}+\tfrac{b_t(x)}{\mu (\varOmega )}\quad \text {for all }x,y\in \varOmega ,\,z\in {\mathbb {R}}_+; \end{aligned}$$

hence, (5.6) is defined on the cone $U{:}{=}C(\varOmega ,{\mathbb {R}}_+)$. If ${\mathbb {I}}$ is unbounded below, then (5.6) possesses a pullback attractor ${\mathcal {A}}^*\subseteq {\mathcal {U}}$; see Fig. 2 for an illustration.

For our subsequent analysis it is convenient to set $\gamma {:}{=}\sup _{x\in \varOmega }\int _\varOmega k(x,y)\,{\mathrm {d}}y$. We begin with some preparatory estimates. Above all, (5.6) satisfies the assumption $(H_4)$ with $\lambda _t(x,y)=\alpha _tk(x,y)$, which guarantees the global Lipschitz condition5.7If we represent the right-hand side of (5.6) in semilinear form (2.5) withthen  holds, as well as the global Lipschitz condition5.8In order to obtain information on the forward attractor, we suppose that ${\mathbb {I}}={\mathbb {Z}}$ and that (5.6) is asymptotically autonomous in forward time, i.e., there exist $\alpha _+>0$ and $b\in C(\varOmega ,{\mathbb {R}}_+)$ such that

$$\begin{aligned} \lim _{t\rightarrow \infty }\alpha _t&=\alpha _+\in [0,1),&\lim _{t\rightarrow \infty }b_t&=b. \end{aligned}$$

If $\alpha _+\gamma <1$, then it follows from the contraction mapping principle and (5.7) that the autonomous limit equation

5.9 $$\begin{aligned} u_{t+1}(x) = \alpha _+ \int _\varOmega k(x,y)u_t(y)e^{-u_t(y)}\,{\mathrm {d}}y+b(x) \quad \text {for all }(t,x)\in {\mathbb {I}}'\times \varOmega \end{aligned}$$

has a unique, globally attractive fixed-point $u^*\in U$.

We choose ${\mathbb {I}}={\mathbb {N}}_0$ and an absorbing set $A\subseteq U$ of the limit equation (5.9) such that ${\mathcal {A}}={\mathbb {I}}\times A$ is forward absorbing w.r.t. (5.6). If we assume $\sup _{s\le t}\prod _{r=s}^{t-1}\frac{\alpha _r}{\alpha _+}\le K$, then the growth estimate (4.13) holds with $\alpha =\alpha _+\gamma$ due toIt follows from (5.8) that every nonlinearity  has the Lipschitz constant $L{:}{=}(1+\tfrac{1}{e^2})\gamma \sup _{t\ge 0}\alpha _t$. Consequently, if furthermore $(\alpha _t)_{t\in {\mathbb {I}}}$ converges exponentially to $\alpha _+$ with rate $\alpha$, then Theorem 4.15 applies under the assumption

5.10 $$\begin{aligned} (1+\tfrac{1}{e^2})\gamma \sup _{t\ge 0}\alpha _t<\tfrac{1-\alpha }{K} \end{aligned}$$

and thus (4.9) holds. Hence, we derive from Theorem 4.11 the relations

$$\begin{aligned} \varOmega _{{\mathcal {A}}}(\tau )=\omega _{\mathcal {A}}^-=\omega _{\mathcal {A}}^+=\left\{ u^*\right\} \quad \text {for all }\tau \in {\mathbb {N}}_0. \end{aligned}$$

If we concretely define

$$\begin{aligned} \alpha _t&{:}{=} {\left\{ \begin{array}{ll} \alpha _+(1+\alpha ^t),&{}t\ge 0,\\ \alpha _-,&{}t<0, \end{array}\right. }&b_t&{:}{=} {\left\{ \begin{array}{ll} b,&{}t\ge 0,\\ 0,&{}t<0, \end{array}\right. } \end{aligned}$$

then this implies the elementary estimate

$$\begin{aligned} \prod _{r=s}^{t-1}\frac{\alpha _r}{\alpha _+} = \exp \bigl (\sum _{r=s}^{t-1}\ln (1+\alpha ^r)\bigr ) \le \exp \bigl (\sum _{r=s}^{t-1}\alpha ^r\bigr ) \le \exp \bigl (\tfrac{1}{1-\alpha }\bigr )\quad \text {for all }s\le t. \end{aligned}$$

Hence, we set $K{:}{=}\exp \bigl (\tfrac{1}{1-\alpha }\bigr )$ and (5.10) simplifies to $2(1+\tfrac{1}{e^2})\alpha \exp \bigl (\tfrac{1}{1-\alpha }\bigr )<1-\alpha$. This assumption can be fulfilled for $\alpha =\alpha _+\gamma$ sufficiently close to 0, which requires the kernel data $\gamma$ or the asymptotic growth rate $\alpha _+$ to be small.

Even more concretely, on the habitat $\varOmega =[-L,L]$ with some real $L>0$ we again consider the Laplace kernel $k(x,y){:}{=}\tfrac{a}{2}e^{-a\left| x-y\right| }$ with dispersal rate $a>0$, which yields $\gamma =1-e^{-aL}$. In this framework, an illustration of the forward limit set $\omega _{\mathcal {A}}^+=\left\{ u^*\right\}$ and subfibres of the pullback attractor ${\mathcal {A}}^*$ is given in Fig. 2. Here, both the pullback attractor ${\mathcal {A}}^*$ and the forward limit set $\omega _{\mathcal {A}}^+$ capture the long term behaviour of (5.6).

## References

[1] Atkinson KE (1992). A survey of numerical methods for solving nonlinear integral equations. J. Integr. Equ. Appl..

[2] Aulbach B, Van Minh N (1996). The concept of spectral dichotomy for linear difference equations II. J. Differ. Equ. Appl..

[3] Bouhours J, Lewis MA (2016). Climate change and integrodifference equations in a stochastic environment. Bull. Math. Biol..

[4] Carvalho AN, Langa JA, Robinson JC (2012). Attractors for Infinite-dimensional Non-autonomous Dynamical Systems, Applied Mathematical Sciences 182.

[5] Cui, H., Kloeden, P.E.: Comparison of attractors of asymptotically equivalent difference equations. In: Elaydi, S., et al. (Eds.) Difference Equations, Discrete Dynamical Systems and Applications, Proceedings of the Mathematical Statistics, Vol. 287, pp. 31–50. Springer, Cham (2019)

[6] Cui H, Kloeden PE, Yang M (2019). Forward omega limit sets of nonautonomous dynamical systems. Discrete Contin. Dyn. Syst. Ser. B.

[7] Day S, Junge O, Mischaikow K (2004). A rigerous numerical method for the global dynamics of infinite-dimensional discrete dynamical systems. SIAM J. Appl. Dyn. Syst..

[8] Hale JK (1988). Asymptotic Behaviour of Dissipative Systems, Mathematical Surveys and Monographs 25.

[9] Jacobsen J, Jin Y, Lewis MA (2015). Integrodifference models for persistence in temporally varying river environments. J. Math. Biol..

[10] Johnson R, Muñoz-Villarragut V (2009). Some questions concerning attractors for non-autonomous dynamical systems Nonlin. Analysis.

[11] Kloeden PE (2000). Pullback attractors in nonautonomous difference equations. J. Differ. Equ. Appl..

[12] Kloeden PE, Lorenz T (2016). Construction of nonautonomous forward attractors. Proc. Am. Math. Soc..

[13] Kloeden PE, Marín-Rubio P (2011). Negatively invariant sets and entire solutions. J. Dyn. Differ. Equ..

[14] Kloeden PE, Pötzsche C, Rasmussen M (2012). Limitations of pullback attractors for processes. J. Differ. Equ. Appl..

[15] Kloeden PE, Rasmussen M (2011). Nonautonomous Dynamical Systems, Mathematical Surveys and Monographs 176.

[16] Kloeden PE, Yang M (2016). Forward attraction in nonautonomous difference equations. J. Differ. Equ. Appl..

[17] Kot M, Schaffer WM (1986). Discrete-time growth-dispersal models. Math. Biosci..

[18] Lutscher F (2019). Integrodifference Equations in Spatial Ecology, Interdisciplinary Applied Mathematics 49.

[19] Martin, R.H.: Nonlinear Operators and Differential Equations in Banach Spaces, Pure and Applied Mathematics 11. Wiley, Chichester (1976)

[20] Pötzsche, C.: Geometric Theory of Discrete Nonautonomous Dynamical Systems, Lecturer Notes in Mathematics, Vol. 2002. Springer, Berlin (2010)

[21] Pötzsche C (2015). Order-preserving nonautonomous discrete dynamics: attractors and entire solutions. Positivity.

[22] Przyłuski K (1988). Remarks on the stability of linear infinite-dimensional discrete-time systems. J. Differ. Equ..

[23] Sell GR, You Y (2002). Dynamics Of Evolutionary Equations, Applied Mathematical Sciences 143.

[24] Vishik MI (1992). Asymptotic Behaviour of Solutions to Evolutionary Equations.

[25] Volkov D, Lui R (2007). Spreading speed and travelling wave solutions of a partially sedentary population. IMA J. Appl. Math..

